# FtsEX-mediated regulation of the final stages of cell division reveals morphogenetic plasticity in *Caulobacter crescentus*

**DOI:** 10.1371/journal.pgen.1006999

**Published:** 2017-09-08

**Authors:** Elizabeth L. Meier, Allison K. Daitch, Qing Yao, Anant Bhargava, Grant J. Jensen, Erin D. Goley

**Affiliations:** 1 Department of Biological Chemistry, Johns Hopkins University School of Medicine, Baltimore, Maryland, United States of America; 2 Division of Biology and Biological Engineering, California Institute of Technology, Pasadena, California, United States of America; 3 Howard Hughes Medical Institute, California Institute of Technology, Pasadena, California, United States of America; Max Planck Institute for Terrestrial Microbiology, GERMANY

## Abstract

During its life cycle, *Caulobacter crescentus* undergoes a series of coordinated shape changes, including generation of a polar stalk and reshaping of the cell envelope to produce new daughter cells through the process of cytokinesis. The mechanisms by which these morphogenetic processes are coordinated in time and space remain largely unknown. Here we demonstrate that the conserved division complex FtsEX controls both the early and late stages of cytokinesis in *C*. *crescentus*, namely initiation of constriction and final cell separation. *ΔftsE* cells display a striking phenotype: cells are chained, with skinny connections between cell bodies resulting from defects in inner membrane fusion and cell separation. Surprisingly, the thin connections in *ΔftsE* cells share morphological and molecular features with *C*. *crescentus* stalks. Our data uncover unanticipated morphogenetic plasticity in *C*. *crescentus*, with loss of FtsE causing a stalk-like program to take over at failed division sites.

## Author summary

Bacterial cell shape is genetically hardwired and is critical for fitness and, in certain cases, pathogenesis. In most bacteria, a semi-rigid structure called the cell wall surrounds the inner membrane, offering protection against cell lysis while simultaneously maintaining cell shape. A highly dynamic macromolecular structure, the cell wall undergoes extensive remodeling as bacterial cells grow and divide. We demonstrate that a broadly conserved cell division complex, FtsEX, relays signals from the cytoplasm to the cell wall to regulate key developmental shape changes in the α-proteobacterium *Caulobacter crescentus*. Consistent with studies in diverse bacteria, we observe strong synthetic interactions between *ftsE* and cell wall hydrolytic factors, suggesting that regulation of cell wall remodeling is a conserved function of FtsEX. Loss of FtsE causes morphological defects associated with both the early and late stages of division. Intriguingly, without FtsE, cells frequently fail to separate and instead elaborate a thin, tubular structure between cell bodies, a growth mode observed in other α-proteobacteria. Overall, our results highlight the plasticity of bacterial cell shape and demonstrate how altering the activity of one morphogenetic program can produce diverse morphologies resembling those of other bacteria in nature.

## Introduction

Bacteria are capable of adopting an impressive array of shapes exquisitely tuned for their particular environmental niches. Underpinning these shapes is the bacterial cell wall, which plays an essential role in specifying and maintaining diverse morphologies [1]. The cell wall consists of a layer of peptidoglycan (PG) composed of glycan strands of repeating disaccharide subunits crosslinked by pentapeptide bridges. In addition to adapting to changing environments, the PG also undergoes dynamic remodeling to drive shape changes during dedicated cellular processes such as division [2,3].

The α-proteobacterium *Caulobacter crescentus* is an ideal model organism for the study of cell shape as it undergoes a series of coordinated morphogenetic changes during its cell cycle. After every division event, *C*. *crescentus* produces two distinct daughter cell types. One is a flagellated, motile swarmer cell, which contains a flagellum and pili at one cell pole [4]. The other is a sessile stalked cell, where the polar flagellum has been replaced by a thin, tubular extension of the cell envelope known as a stalk [4]. Unable to replicate its chromosome or initiate cell division, a swarmer cell differentiates into a stalked cell by ejecting its flagellum, disassembling its pili, and growing a stalk at the same pole [4]. A stalked cell then elongates its cell body, replicates and segregates its DNA, and produces a flagellum at the pole opposite its stalk prior to cytokinesis. The asymmetric polarization of distinct organelles imparts *C*. *crescentus* with a highly tractable dimorphic life cycle ideally suited for studying developmental shape changes.

One cell cycle event that requires obvious reshaping of the cell envelope is cell division, which, in nearly all bacteria, requires the conserved tubulin homolog FtsZ. A GTPase, FtsZ polymerizes into a patchy annular structure (the Z-ring) at the incipient division site and recruits the downstream division machinery or divisome. Together, FtsZ and proteins of the divisome coordinate invagination and fission of the membrane(s) with extensive cell wall remodeling [5]. A number of division proteins are known to interact directly with FtsZ. For many of these regulators, however, their mechanism of action toward FtsZ and physiological role in division remain to be discovered. One division complex that has been particularly enigmatic is the ATP-binding cassette (ABC) transporter family complex FtsEX, which is widely conserved in bacteria and functions as a heterodimer with FtsE in the cytoplasm and FtsX in the inner membrane. In *Escherichia coli*, FtsEX localizes to the septum and contributes to the efficiency of cell division, particularly in salt-free media [6]. There is no evidence that FtsEX acts as a transporter, however, and recent studies from a wide range of bacteria have instead implicated FtsEX in the activation of cell wall hydrolysis [7–11]. Septal PG material needs to be split to allow for outer membrane constriction and ultimately for separation of the two new daughter cells. In high salt media, *E*. *coli ΔftsEX* cells exhibit a phenotype similar to cell wall hydrolysis mutants, i.e. cells are chained and mildly filamentous, suggesting a common genetic pathway [9].

In addition to its involvement in cell wall hydrolysis, in *E*. *coli*, FtsEX is important for the recruitment of late division proteins and the assembly and/or stability of the septal ring [6]. Interestingly, *E*. *coli ftsE* mutants impaired for ATP binding and hydrolysis support Z-ring assembly, but constrict poorly [12]. Since FtsE interacts with FtsZ in *E*. *coli*, one possibility is that FtsEX functions as a membrane anchor for FtsZ and utilizes ATP binding and hydrolysis to regulate Z-ring constriction [13,6].

In this study, we were originally motivated to characterize FtsEX as a novel membrane anchor for FtsZ in *C*. *crescentus* since FtsE is one of the first proteins recruited to the nascent division site and is important for efficient cell separation and Z-ring assembly and/or stability [14,15]. Considering the conserved function of FtsEX as a modulator of cell wall remodeling, we asked whether FtsEX, in addition to promoting Z-ring structure, regulates cell wall cleavage in *C*. *crescentus*. We find that *ftsE* has strong synthetic cell separation defects with cell wall hydrolytic factors. Interestingly, however, deleting *ftsE* produces chains of cell bodies connected by thin, tube-like connections that contain all layers of the cell envelope. This is in stark contrast to the thick, uncleaved septa and compartmentalized cytoplasms observed in hydrolysis mutants from *E*. *coli* and other organisms. The cell-cell connections of Δ*ftsE* cells are, instead, morphologically and topologically similar to *C*. *crescentus* stalks. In accordance with their shared morphological features, the stalk proteins StpX and PbpC localize to both the skinny connections and stalks of *ΔftsE* cells, indicating that stalk formation may be mechanistically similar to the elaboration of the extended constriction sites. Our data reveal unanticipated morphogenetic plasticity in *C*. *crescentus*, with a stalk-like program taking over at failed division sites in the Δ*ftsE* mutant.

## Results

### *ΔftsE* cells form chains connected by skinny constrictions

**Fig 1:**
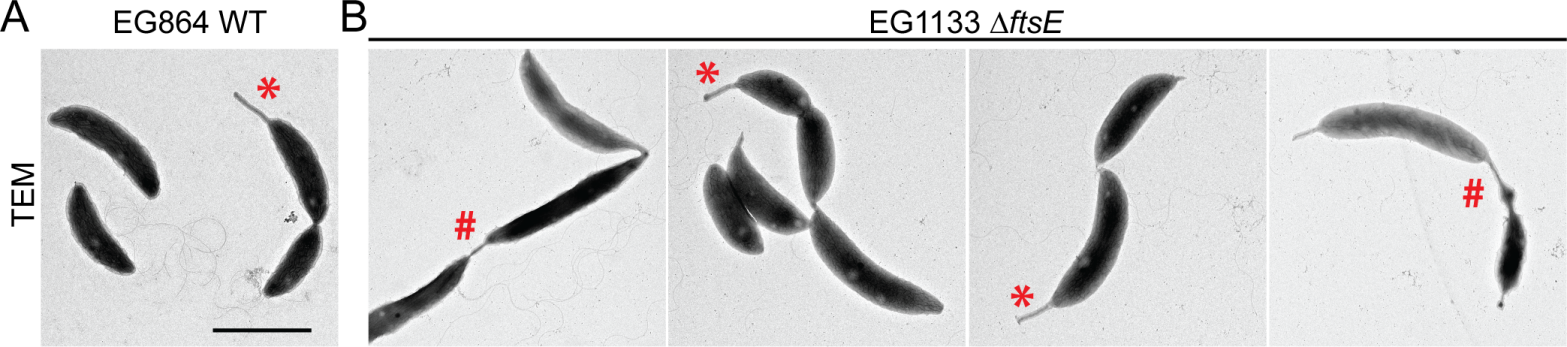
Transmission electron microscopy (TEM) of chained *ΔftsE* cells reveals thin, extended connections between cell bodies. (A) Micrograph of WT cells. (B) Micrographs of *ΔftsE* cells. * = stalk; # = skinny connection. Scale bar = 2 μm.

To begin to address the role of FtsEX in *C*. *crescentus*, we first attempted to make *ftsE* and *ftsX* deletion strains. Although *ftsE* is annotated as essential [14,16], we readily obtained several independent *ΔftsE* clones, particularly when we focused on isolating smaller colonies after sucrose counterselection [15]. *ftsX* is also annotated as essential [16], but unlike *ftsE*, we were unable to make an *ftsX* deletion, depletion, or overexpression strain, suggesting that *C*. *crescentus* cells are highly sensitive to changes in FtsX levels. The difference in essentiality of *ftsE* and *ftsX* suggests that FtsX fulfills an FtsE-independent function that is required for viability. To understand the role of the FtsEX complex in *C*. *crescentus* morphogenesis, we focused on characterizing the *ftsE* mutant in detail. Using phase contrast microscopy, we previously reported that *ΔftsE* cells displayed a striking division phenotype consisting of chained cells with skinny connections between cell bodies [15]. Transmission electron microscopy (TEM) offered us better resolution of cells lacking FtsE and highlighted two prominent features of the cell division defects in Δ*ftsE* cells (Fig 1). First, *ΔftsE* cell bodies were heterogeneous in length, but overall appeared elongated compared to WT, which suggests a delay or inefficiency in the initiation of constriction. Second, in chained *ΔftsE* cells, the cell bodies were joined by tube-like connections that were thin and heterogeneous in length, with some extending hundreds of nanometers (Fig 1B). Consistent with the chaining phenotype, *ΔftsE* cells are longer and grow more slowly than WT [15]. Overall, the *ΔftsE* phenotype supports a role for FtsE both in the initiation of constriction and in late stage cell separation. It is important to note that the *ΔftsE* phenotype may not entirely be due to loss of FtsE; an unregulated activity of FtsX might occur when FtsX is no longer in complex with FtsE and contribute to the observed phenotypes. We will first describe our characterization of FtsE’s role in Z-ring assembly and the initiation of constriction before addressing FtsE’s involvement in the final stages of cell separation.

### FtsE promotes focused Z-ring organization

**Fig 2:**
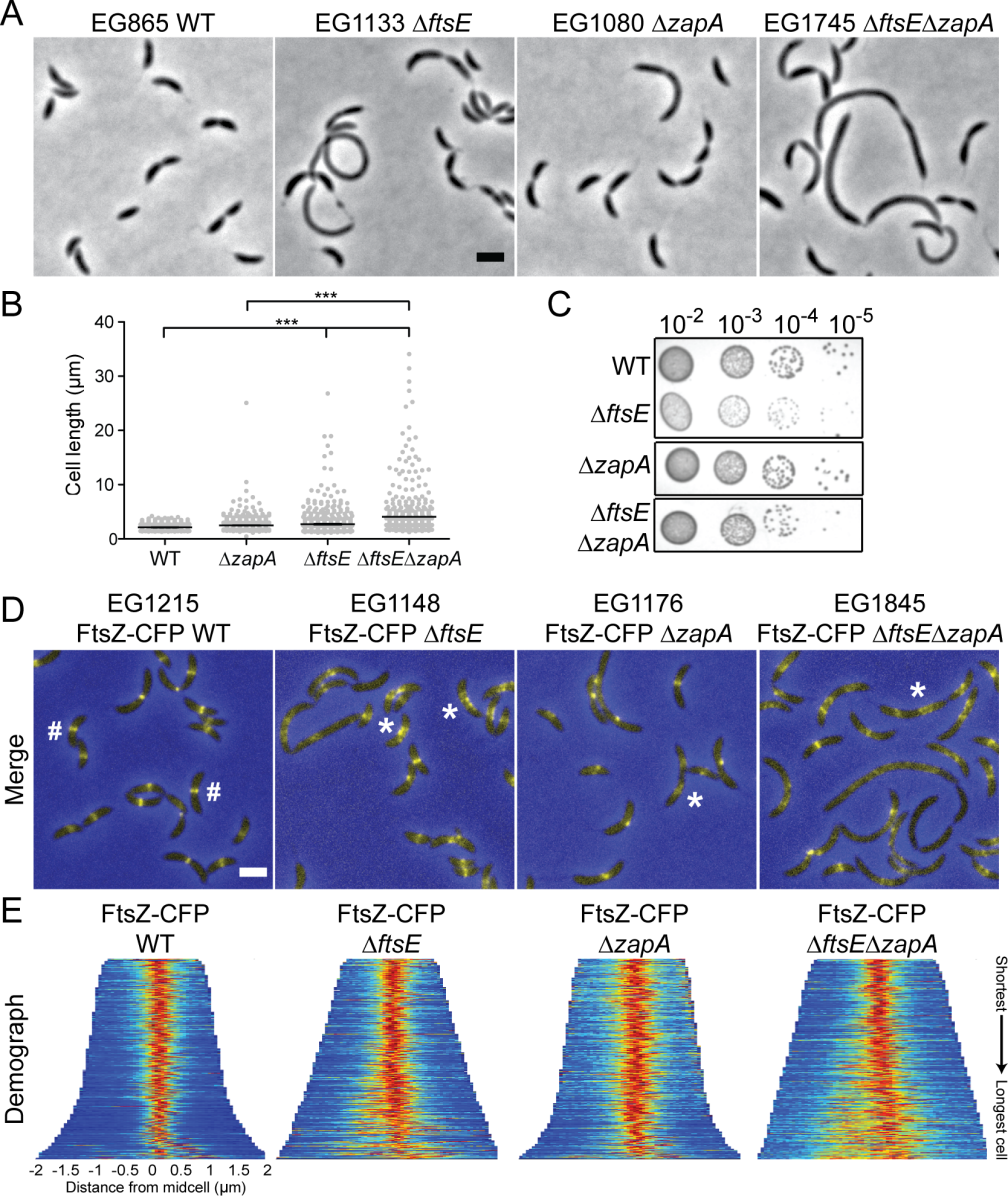
*ftsE* has synthetic cell length, growth, and Z-ring structural defects with Z-ring regulator *zapA*. (A) Phase contrast micrographs of WT, *ΔftsE*, *ΔzapA*, and *ΔftsEΔzapA* cells. (B) Cell length analyses of strains in (A). Mean cell length (μm) ± SEM: EG865 = 2.12 ± 0.02 (N = 500); EG1080 = 2.48 ± 0.04 (N = 779); EG1133 = 2.7 ± 0.07 (N = 747); EG1745 = 4.10 ± 0.18 (N = 500). *** = p value < 0.001 by one-way ANOVA. (C) Spot dilutions of strains in (A). Cells in log phase were diluted to an OD_600_ of 0.05, serially diluted and spotted onto the same PYE agar plate, and incubated at 30°C for 2 days. Images were cropped as shown and re-ordered for clarity of presentation. (D) FtsZ-CFP localization in merged fluorescence (yellow) and phase contrast (blue) micrographs after 1 h of induction in WT, *ΔftsE*, *ΔzapA*, and *ΔftsEΔzapA* cells. # = focused Z-ring; * = diffuse, punctate Z-ring. (E) Demographs of FtsZ-CFP localization in strains in (D). Cells were arranged from shortest to longest. N = 400 for each strain and maximum cell length was limited to 4 μm to disregard effects of filamentation on Z-ring organization. Scale bars = 2 μm.

FtsE has been reported to bind FtsZ in *E*. *coli*, is one of the first division proteins to localize to midcell after FtsZ in *C*. *crescentus*, and *C*. *crescentus ΔftsE* cells have aberrant Z-rings [13–15]. Specifically, in *ΔftsE* cells FtsZ is more diffuse and often localizes as clusters of puncta instead of focused Z-rings (Fig 2D) [15]. These data suggest that FtsE may regulate Z-ring structure and/or assembly and thereby contribute to efficient initiation of constriction. Consequently, we tested if *ftsE* interacted genetically with the *zapA* gene encoding the positive Z-ring regulator ZapA. Like FtsE, ZapA is also recruited early to midcell by FtsZ in *C*. *crescentus* and contributes to proper organization of FtsZ at midcell and efficient division [14,17–18]. *ΔzapAΔftsE* cells displayed a synthetic cell division defect, as evidenced by the increased filamentation observed in the double mutant as compared to either single mutant, and had severely disrupted, diffuse Z-ring structures (Fig 2). Midcell rings of FtsZ were rare in the double mutant and, when present, were more diffuse than WT (Fig 2D and 2E). We conclude that FtsE contributes to proper Z-ring assembly at midcell and that the elongated cell bodies in *ΔftsE* are likely due to inefficient initiation of constriction by the aberrant FtsZ structures.

### Excess FtsE or FtsEX alters the localization of FtsZ and new cell wall synthesis

**Fig 3:**
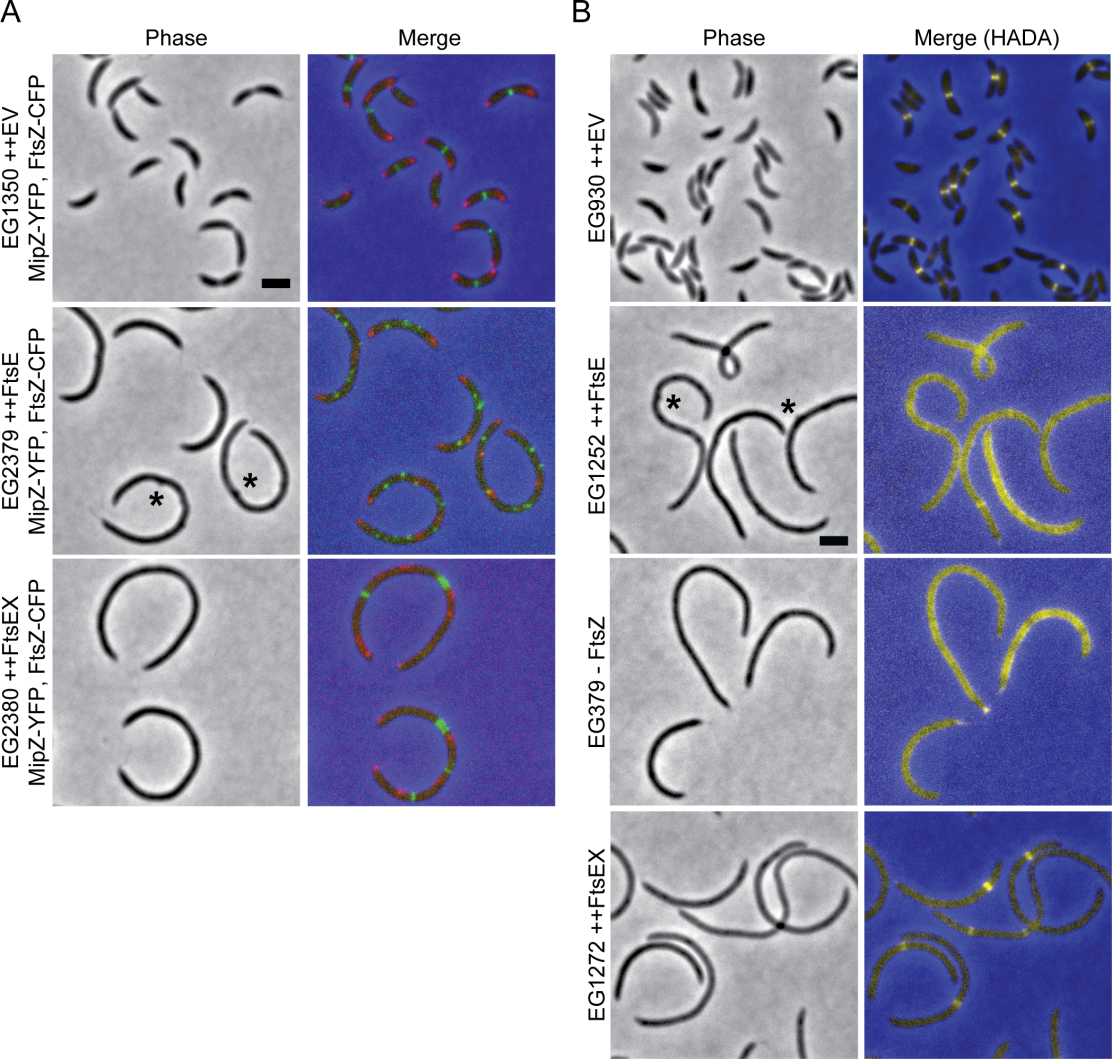
Overproducing FtsE or FtsEX causes filamentation and affects the localization of FtsZ and new PG. (A) FtsZ-CFP (green) and MipZ-YFP (red) localization after 1 h of induction in cells with *mipZ-yfp* at the native *mipZ* locus bearing an empty vector (EV) or overexpressing *ftsE* or *ftsEX* for 4 h. (B) HADA labeling of cells depleted of FtsZ for 4 h, bearing an EV, or overexpressing *ftsE* or *ftsEX* for 4 h. * = envelope bulge. Scale bars = 2 μm.

Because cells lacking FtsE have perturbed Z-ring organization, we hypothesized that overproducing FtsE would affect Z-ring structure, particularly if FtsE binds directly to FtsZ. Overexpression of either *ftsE* or *ftsEX* caused dramatic filamentation, and overexpression of *ftsE* alone also caused small, local cell envelope bulges to form (Fig 3A). After four hours of FtsE overproduction, instead of Z-rings, FtsZ-CFP formed discrete puncta along the length of the filamentous cells (Fig 3A). Interestingly, when we overproduced FtsEX, FtsZ-CFP localized in a drastically different pattern, as multiple rings or wide bands (Fig 3A). *C*. *crescentus* Z-ring positioning is in part dictated by a negative regulator of FtsZ assembly called MipZ, which forms a complex near the origin of replication [19]. After the polar origin region is duplicated, the second copy is quickly transported to the opposite cell pole. Bipolar MipZ thereby directs Z-ring assembly at midcell by inhibiting FtsZ polymerization at the poles [19]. To assess FtsZ’s localization relative to MipZ, we visualized natively tagged MipZ-YFP in FtsZ-CFP producing cells overexpressing *ftsE* or *ftsEX*. MipZ-YFP localized at the poles and as fairly regularly spaced puncta in cells overproducing FtsE or FtsEX, but its localization was more diffuse in cells overproducing FtsEX (Fig 3A). We did not observe colocalization of FtsZ-CFP or MipZ-YFP in *ftsE* or *ftsEX* overexpressing cells, but frequently in *ftsEX* overexpressing cells broad bands of FtsZ localized directly adjacent to regions enriched for MipZ, suggesting that these wide bands of FtsZ may be more resistant to MipZ inhibition than WT Z-rings. We interpret the MipZ localization data as evidence that chromosome replication and segregation still occur in cells overproducing FtsE despite the inhibition of division; however high levels of FtsEX may interfere with levels or localization of MipZ.

In *C*. *crescentus*, FtsZ directs new cell wall synthesis at midcell even before the onset of division (Fig 3B) [20]. To determine if the FtsZ structures in FtsE or FtsEX overproducing cells were competent to localize new cell wall synthesis, we pulse-labeled cells with the fluorescent D-amino acid hydroxycoumarin-carbonyl-amino-D-alanine (HADA) to track the incorporation of newly synthesized PG [21]. High levels of FtsE resulted in diffuse HADA labeling which closely resembled the pattern of HADA incorporation in FtsZ-depleted cells (Fig 3B). This result suggests that the FtsZ puncta associated with *ftsE* overexpression are unable to direct local cell wall metabolism. Cells overexpressing *ftsEX*, however, had discrete, wide bands of new PG incorporation, likely directed by the similarly organized Z-ring structures in these cells (Fig 3B). Thus, high levels of FtsE or FtsEX not only differentially affect Z-ring organization, but also affect FtsZ’s ability to locally direct new cell wall synthesis. Specifically, the intact FtsEX complex is required both for formation of Z-rings and downstream communication with PG synthetic machinery. The broad Z-rings in FtsEX-overproducing cells are not, however, competent to drive envelope constriction.

### *ftsE* interacts genetically with PG hydrolytic factors and a regulator of stalked pole development

The aberrant Z-rings formed in Δ*ftsE* cells likely explain the cell body elongation observed in the mutant. We next turned our attention to the cell chaining aspect of the Δ*ftsE* phenotype. FtsEX has been implicated in cell wall hydrolysis, which is required for daughter cell separation at the end of division, in numerous bacteria. We therefore asked whether FtsEX, in addition to promoting Z-ring structure, regulates *C*. *crescentus* cell wall hydrolysis. In *E*. *coli*, periplasmic *N*-acetylmuramyl-_L_-alanine amidases AmiA/B/C are responsible for cleaving bonds that link stem peptides to glycan strands at the septum [22]. The amidases require activation by the LytM domain containing proteins EnvC, which stimulates AmiA/B, and NlpD, which stimulates AmiC, to split apart septal PG [23–24]. FtsEX directly recruits EnvC to the septum via the periplasmic extracellular loop (ECL) of FtsX and the coiled coil (CC) domain of EnvC [9]. *C*. *crescentus* possesses a limited number of lytic enzymes involved in peptidoglycan remodeling, with only a single *N*-acetylmuramyl-_L_-alanine amidase, most similar to *E*. *coli* AmiC [25–26]. There are at least seven putative LytM domain containing proteins; however, the only characterized protein in the *C*. *crescentus* LytM family is DipM, which participates in cell wall remodeling and coordinated constriction of the cell envelope layers at the division plane [26–28]. To determine whether the FtsEX PG hydrolysis paradigm applies to *C*. *crescentus*, we performed a BLAST search for *E*. *coli* EnvC homologues and found CCNA_03547, which we will hereafter refer to as *L*ytM *d*omain *p*rotein *F* (LdpF) (Martin Thanbichler, personal communication). Like EnvC, LdpF is a LytM domain containing protein with a signal peptide, two N-terminal CC domains, and a C-terminal LytM domain (S1A Fig). We hypothesized that *C*. *crescentus* FtsEX-LdpF-AmiC may function in an activation pathway analogous to *E*. *coli* FtsEX-EnvC-AmiA/B.

**Fig 4:**
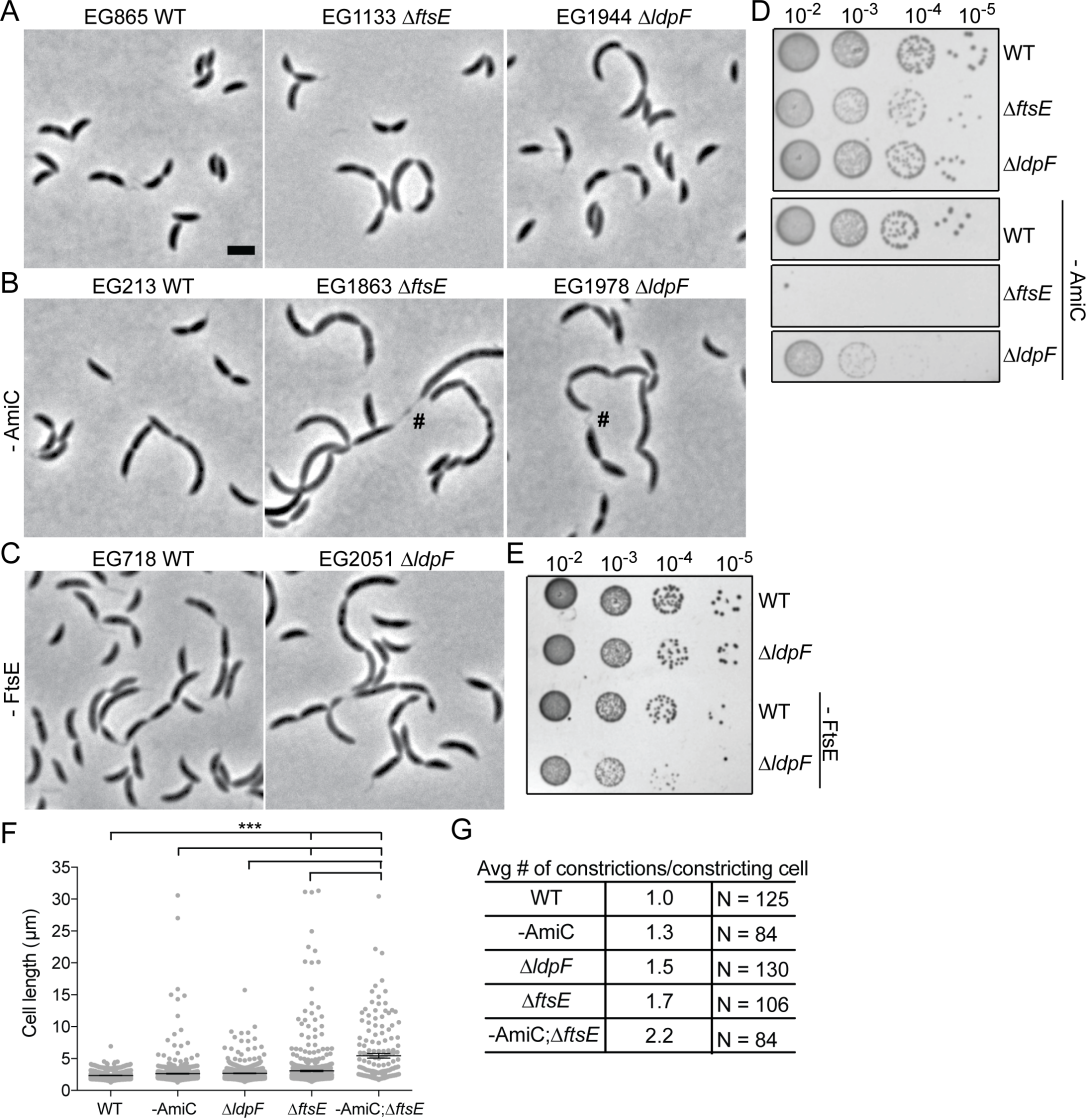
Synthetic genetic interactions are observed between *amiC*, *ftsE*, and *ldpF*. Phase contrast micrographs of (A) WT, *ΔftsE*, or *ΔldpF* cells; (B) WT, *ΔftsE*, or *ΔldpF* cells depleted of AmiC for 19 h; (C) WT or *ΔldpF* cells depleted of FtsE for 19 h. # = highly elongated skinny connections. Scale bar = 2 μm. (D,E) Spot dilutions of strains in (A-C). Depletion strains were grown for 19 h without inducer before spotting on PYE agar plates also without inducer. Cells in log phase were diluted to an OD_600_ of 0.05, serially diluted and spotted onto PYE agar plates, and incubated at 30°C for 2 days. Strains from (D) were spotted on the same PYE agar plate and strains from (E) were spotted on the same PYE agar plate. Images were cropped when necessary as shown and re-ordered for clarity of presentation. (F) Cell length distributions of the indicated strains. Mean ± SEM is indicated. Mean cell length (μm) ± SEM: WT = 2.34 ± 0.02 (N = 737); -AmiC = 2.65 ± 0.06 (N = 800); *ΔldpF* = 2.69 ± 0.04 (N = 797); *ΔftsE* = 3.10 ± 0.11 (N = 722); -AmiC;*ΔftsE* = 5.44 ± 0.37 (N = 159). *** = p value < 0.001 by one-way ANOVA. Depletion strains were grown for 24 h without inducer. (G) Number of constrictions in visibly constricting cells from strains in (F) were manually counted and divided by the total number of constricting cells to calculate the average number of constrictions per constricting cell.

We first adopted a genetic approach to investigate the role of FtsEX in cell wall hydrolysis during division. In *E*. *coli*, FtsEX is required for EnvC’s localization at midcell [9]. However, a functional LdpF-mCherry fusion is diffuse in the cell periphery, and we did not observe differences in its localization between WT and *ΔftsE* cells. LdpF-mCherry levels were lower in *ΔftsE* compared to WT, however (S1B–S1D Fig). Although *E*. *coli* cells lacking EnvC or AmiA/B are not as sick as cells lacking FtsEX, part of the division defect associated with loss of FtsEX may be due to EnvC inactivation [9]. Consistent with this reasoning, in *C*. *crescentus*, cells lacking LdpF or AmiC had mild cell chaining, length, and growth defects compared to *ΔftsE* cells (Fig 4A, 4B, 4D, 4F and 4G). Depleting AmiC in Δ*ftsE* or Δ*ldpF* backgrounds, however, caused strong synthetic cell chaining, length, and growth defects, accompanied by noticeable lengthening of the skinny connections between cell bodies, particularly in the *ftsE* mutant background (Fig 4B, 4D, 4F and 4G). Combining *ΔldpF* with loss of FtsE produced more moderate synthetic growth and chaining defects, which is consistent with them acting in a common pathway (Fig 4C and 4E).

**Fig 5:**
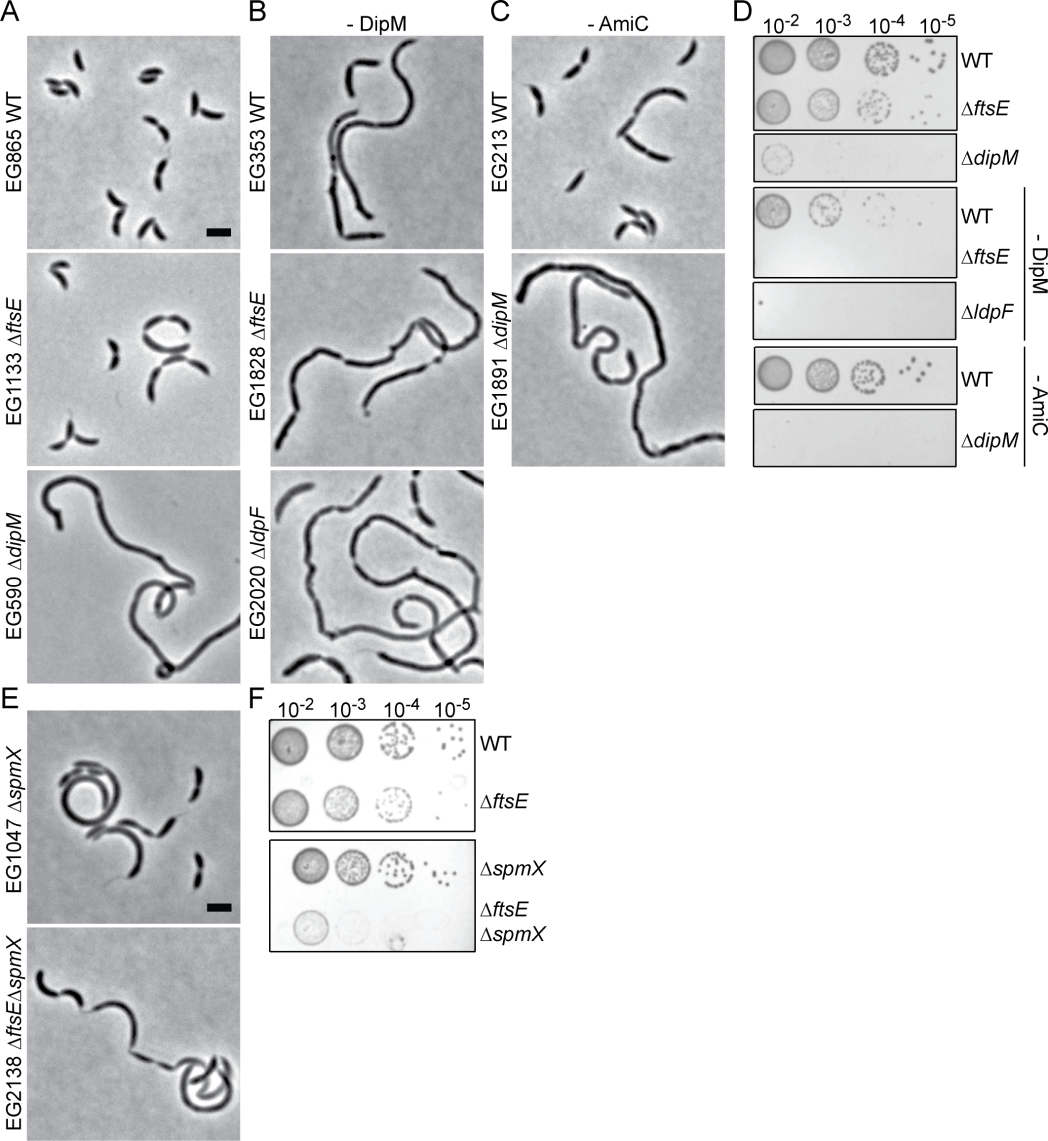
Synthetic genetic interactions are observed between *dipM* and *amiC*, *ftsE*, or *ldpF* and between *ftsE* and *spmX*. Phase contrast micrographs of (A) WT, *ΔftsE*, and *ΔdipM* cells; (B) WT, *ΔftsE*, or *ΔldpF* cells depleted of DipM for 19 h; (C) WT or *ΔdipM* cells depleted of AmiC for 19 h. (D) Spot dilutions of strains in (A-C). Depletion strains were grown for 19 h without inducer before spotting on PYE agar plates also without inducer. Cells in log phase were diluted to an OD_600_ of 0.05, serially diluted and spotted onto the same PYE agar plate, and incubated at 30°C for 2 days. Images were cropped as shown and re-ordered for clarity of presentation. (E) Phase contrast micrographs of *ΔspmX* and *ΔftsEΔspmX* cells. (F) Spot dilutions of strains in (E). Cells in log phase were diluted to an OD_600_ of 0.05, serially diluted and spotted onto the same PYE agar plate, and incubated at 30°C for 2 days. Images were cropped as shown and re-ordered for clarity of presentation. Scale bars = 2 μm.

*E*. *coli* cells lacking EnvC depend on NlpD for cell separation: simultaneous inactivation of either EnvC or FtsEX and NlpD results in severe chaining, supporting the hypothesis that FtsEX activates EnvC’s ability to promote septal PG cleavage [23]. In *C*. *crescentus*, the LytM protein most closely related to NlpD is DipM, which has similar domain organization to NlpD but differs in that it is not associated with the outer membrane. Depleting AmiC in cells lacking DipM or depletion of DipM in Δ*ftsE* or Δ*ldpF* cells was synthetic lethal (Fig 5A–5D). The severe morphological and growth defects associated with loss of DipM, alone, complicate the interpretation of synthetic interactions. However, the synthetic lethality associated with loss of DipM and FtsE, LdpF, or AmiC as well as the distinct morphology of *ΔdipM* cells [26–28] suggest that DipM operates in a distinct hydrolytic pathway. We also hypothesize, based on the strong synthetic interactions between AmiC and FtsE or LdpF but only moderate synthetic defects associated with loss of FtsE and LdpF, that AmiC operates in a hydrolytic pathway separate from FtsE and LdpF. However, it is apparent that all three putative PG hydrolytic pathways contribute to the efficiency of cell separation in *C*. *crescentus*.

Interestingly, in a transposon deep-sequencing analysis to uncover transposon insertions that alter the competitive fitness of *ΔspmX* cells, the *ftsE* and *ldpF* loci were the first and second least favored insertion sites, respectively, in *ΔspmX* cells as compared to WT [29]. We therefore tested for synthetic interactions between *ftsE* and *spmX*, which encodes a muramidase homolog that localizes to the stalked cell pole and controls the swarmer-to-stalk cell transition [30]. Consistent with the *ΔspmX* transposon deep-sequencing results, we observed severe synthetic growth and morphological defects for *ΔftsEΔspmX* cells, implicating FtsE and LdpF in the stalked pole development pathway, likely through a connection to cell wall remodeling (Fig 5E and 5F).

### The CC domain of LdpF interacts with the ECL of FtsX, but LdpF does not activate AmiC PG hydrolysis *in vitro*

To provide biochemical support for our *in vivo* findings, we purified LdpF, AmiC, DipM, and the ECL of FtsX, and monitored PG degradation using an *in vitro* dye release assay [24,31]. Bacterial two-hybrid analysis showed a positive interaction for the ECL of FtsX and the CC domain of LdpF, however we did not observe LdpF-activated AmiC PG hydrolysis *in vitro* (S2 Fig; S1 Text). Interestingly, the LytM domain of DipM was sufficient to mildly stimulate AmiC hydrolase activity *in vitro* (S2C Fig; S1 Text). However, as *C*. *crescentus* DipM and AmiC are most similar to the *E*. *coli* activator-amidase pair, NlpD and AmiC, perhaps this result is unsurprising. In light of the distinct phenotypes of cells lacking DipM and AmiC, however, we hypothesize that DipM has activities in addition to the regulation of AmiC. Collectively, our genetic and biochemical evidence indicate at least three hydrolytic pathways in *C*. *crescentus*: (1) FtsEX-LdpF and a yet unidentified downstream target, (2) AmiC, and (3) DipM and a yet unidentified downstream target.

### Electron cryotomography reveals unique cell envelope organization at chaining sites

**Fig 6:**
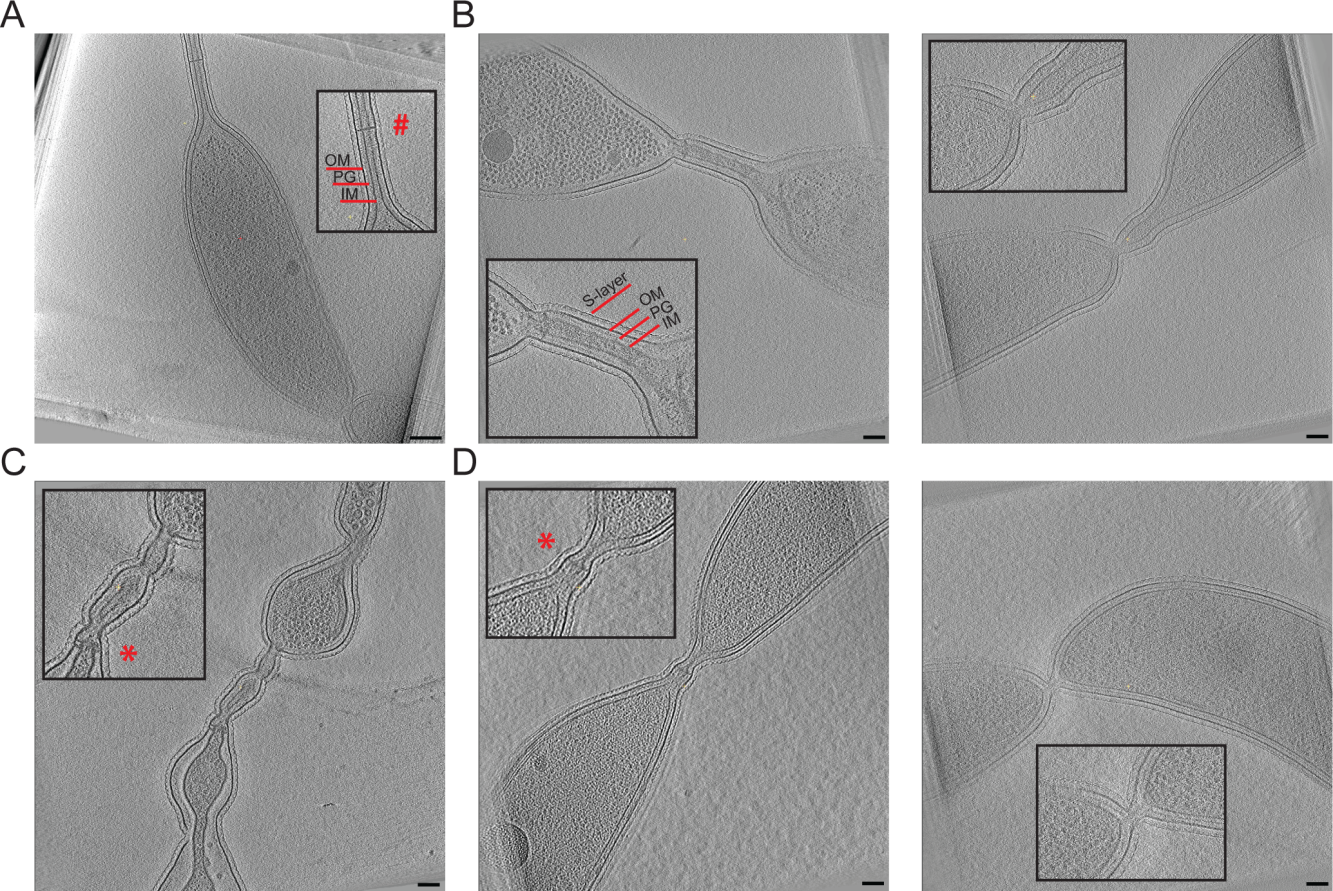
Electron cryotomography (ECT) of cells lacking FtsE and AmiC reveal stalk-like connections between cell bodies. (A-D) Tomogram slices of WT or -AmiC; Δ*ftsE* cells. (A) Late pre-divisional WT *C*. *crescentus* cell highlighting its stalk with cross-band. (B) -AmiC; Δ*ftsE* cells with skinny connections most similar to stalks. (C) -AmiC; Δ*ftsE* cell with a skinny connection that is stalk-like, but that has regions with heterogeneous widths. (D) -AmiC; Δ*ftsE* cells with skinny connections that are fairly short with inner membranes that are close together or nearly fused. Boxed insets are magnifications of specific features from each image. Abbreviations are as follows: OM = outer membrane, PG = peptidoglycan, IM = inner membrane. # = cross-band; * = cross-band-like structure. Scale bar (A) = 200 nm; Scale bars (B-D) = 100 nm.

Our genetic evidence is consistent with a role for FtsEX in regulating cell wall hydrolysis for cell separation. Although TEM highlighted the general cell separation defects of *ΔftsE* cells (Fig 1), electron cryotomography (ECT) allowed us to dissect the exact stages at which these cells are blocked during division (Fig 6). To capture the cell envelope organization at the skinny cell-cell connections, we imaged Δ*ftsE* cells depleted of AmiC (referred to as -AmiC; Δ*ftsE*) since loss of AmiC exacerbates the *ΔftsE* chaining phenotype (Fig 4). In five out of six tomograms, we could identify with certainty the presence of all layers of the cell envelope in the skinny connections between chained cell bodies (Fig 6). Four of these had obvious cytoplasmic volume between the unfused inner membranes. In at least one example, however, the inner membranes were closely stacked on top of each other, but not fused (Fig 6D).

In WT *C*. *crescentus*, the final stages of inner membrane fission are rapid, and the smallest diameter for inner membrane connections (i.e. the distance from inner membrane to inner membrane in a tomogram slice) that have been reported by ECT are ~60 nm [32]. In cells lacking FtsE and AmiC with fairly uniform connections (Fig 6B and 6D), we observed inner membrane connection diameters ranging from ~12 to 60 nm. Others were more variable and had intermittent bulging, with inner membrane connection diameters ranging from ~20 to 300 nm within a single cell-cell connection (Fig 6C, S3A Fig). This organization of the cell envelope is strikingly different from other mutants deficient in cell wall hydrolysis. *E*. *coli* cells lacking EnvC and NlpD or all four LytM-domain containing factors complete inner membrane fusion and cytoplasmic compartmentalization, but struggle to constrict their outer membrane due to a layer of intact PG between adjacent chained cells [23]. Similarly, *ΔdipM* cells form chains with fused inner membranes and thick, multilayered PG between cell bodies [26–27]. Therefore, the cell envelope organization of cells lacking FtsE and AmiC, namely the continuous cytoplasmic connections and narrow spacing between the inner membranes, represents a unique cell separation phenotype and, potentially, a novel pathway for cell separation in *C*. *crescentus*.

### *ΔftsE* thin connections are morphologically stalk-like and enriched for stalk proteins

During its dimorphic life cycle, *C*. *crescentus* elaborates a polar stalk, a tubular extension of the cell envelope important for nutrient uptake [33]. ECT of cells lacking FtsE and AmiC reinforced an observation we had previously made based on TEM of *ΔftsE*, namely, the striking morphological similarities between the extended connections of *ftsE* mutants and *C*. *crescentus* stalks (Figs 1 and 6). In addition to sharing approximate widths (~12–300 nm inner membrane connection diameter for the skinny connections; ~20–40 nm inner membrane connection diameter for the stalks) and cell envelope organization, we occasionally observed electron dense structures that spanned the short axis of the cell envelope of the thin connections (Fig 6C and 6D). These structures were reminiscent of stalk cross-bands, multiprotein assemblies that transect the stalk at regular intervals and function as diffusion barriers to compartmentalize stalk and cell body periplasmic and membrane proteins (Fig 6A, 6C and 6D, S3 Fig) [34].

Since stalk growth occurs by incorporation of new material at the cell body-stalk junction [33], we monitored HADA incorporation at the extended constrictions of the *ftsE* mutants. We observed cells with incorporation of new cell wall material at the base of the skinny connections or only in the cell body, but also often observed cells with HADA incorporation throughout the skinny connections (S4B and S4C Fig). This contrasts with the pattern of *de novo* PG synthesis only at the base of WT *C*. *crescentus* stalks. There are two known modes of zonal PG synthesis in *C*. *crescentus*: FtsZ-independent PG incorporation at the base of the stalk and FtsZ-dependent PG incorporation at midcell [20]. Interestingly, in *ΔftsE* cells, FtsZ rarely localizes throughout the skinny connections, suggesting that the new cell wall synthesis occurring throughout the thin constrictions may be an FtsZ-independent process similar to PG synthesis at the base of the stalk (S4D Fig).

**Fig 7:**
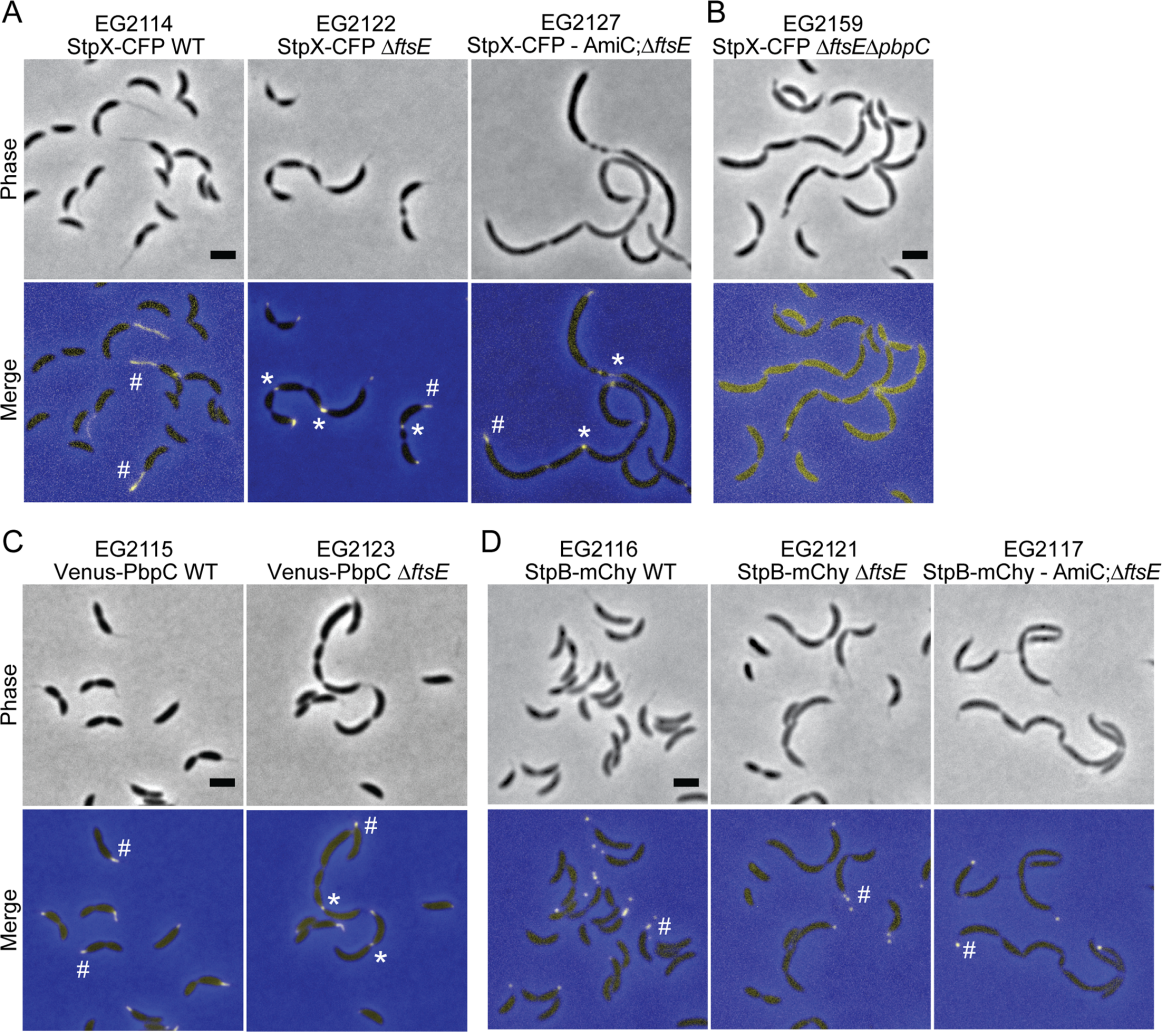
Stalk-localized proteins StpX and PbpC localize to the skinny constrictions in *ftsE* mutants. (A) Localization of StpX-CFP induced for 18 h in WT, *ΔftsE* or *ΔftsE* cells depleted of AmiC for 18 h. (B) Localization of StpX-CFP induced for 2.75 h in *ΔftsEΔpbpC*. (C) Localization of Venus-PbpC induced for 2 h in WT or *ΔftsE*. (D) Localization of cross-band protein StpB-mCherry induced for 18 h in WT, *ΔftsE* or *ΔftsE* cells depleted of AmiC for 18 h. # = stalk enrichment; * = skinny connection enrichment. Scale bars = 2 μm.

Motivated by the morphological similarities between the extended constrictions in *ΔftsE* cells and *C*. *crescentus* stalks, we asked if any stalk-specific proteins could localize to the skinny connections. StpX is a bitopic membrane protein enriched in the stalk that regulates stalk length [35]. We expressed *stpX-cfp* in WT, *ΔftsE*, and AmiC-depleted Δ*ftsE* cells (-AmiC; *ΔftsE*): StpX-CFP localized to the stalks in all genetic backgrounds, but strikingly, was also enriched at the skinny constrictions in the *ftsE* mutant cells (Fig 7A). The bifunctional penicillin binding protein, PbpC, is also involved in stalk elongation [36] and is required to sequester StpX at the stalk in WT *C*. *crescentus* [37]. To investigate the localization dependency of StpX to the skinny constrictions, we expressed *stpX-cfp* in a *ΔftsEΔpbpC* mutant background. Consistent with previous reports, StpX-CFP was not enriched in stalks in cells lacking both FtsE and PbpC. However, StpX-CFP was also not enriched at the skinny connections between cells and instead was diffusely localized at the cell periphery (Fig 7B). We also expressed *venus-pbpC* in WT and *ΔftsE* cells and observed enrichment at the base of stalks as well as frequent localization to the skinny connections in the *ftsE* mutant (Fig 7C). Since we observe enrichment of both PbpC and StpX at the skinny connections in *ΔftsE* cells, we hypothesize that PbpC initially recruits StpX to the skinny connections and promotes its retention at those sites.

Considering the presence of both PbpC and StpX at the skinny connections in the *ftsE* mutant, we monitored the localization of the stalk cross-band protein, StpB. StpB-mCherry localized as puncta in the stalks of WT, *ΔftsE*, and -AmiC; *ΔftsE* cells, but was not enriched at the extended constriction sites (Fig 7D). Consequently, the envelope-spanning discs observed in the *ftsE* mutant by ECT (Fig 6) may not, in fact, be cross-bands or may differ in molecular composition from stalk cross-bands. We conclude that the skinny connections share numerous morphological and molecular similarities with stalks, but the two structures are not physically or biochemically identical.

### MreB inhibition influences the width and length, but not formation, of Δ*ftsE* thin connections

While specialized protein machineries exist for cell division and cell elongation, there is no single protein, much less entire machinery, absolutely and specifically required for stalk formation. The cell elongation machinery proteins MreB and RodA contribute to stalk growth and morphogenesis, however, so stalk synthesis has been proposed to be a specialized form of cell elongation [38]. Thus, we hypothesized that the skinny, stalk-like connections between cell bodies in *ΔftsE* were a result of a modified form of cell elongation, similar to what has been postulated for stalks [38]. In WT and *ΔftsE* cells, Venus-MreB localized in a patchy pattern along the length of the cell bodies and in a ring-like structure at midcell, with only occasional localization at the junction between a skinny connection and a cell body in Δ*ftsE* cells (S6A Fig) [39].

Though MreB was not specifically enriched at the skinny connections in *ΔftsE* cells, we reasoned that MreB still may be relevant to their formation by dynamically localizing or initially positioning the cell wall elongation machinery at those sites. We therefore asked whether inactivation of MreB affects the morphology or formation of the extended constrictions similar to what has been described for *C*. *crescentus* stalks [38]. Addition of the MreB inhibitor MP265, a less toxic A22 structural analog, at the previously published concentrations of 5 or 250 μM to WT or *ΔftsE* cells partially or completely, respectively, inhibited growth and delocalized Venus-MreB (S6B and S6C Fig) [40,41]. Treatment of WT or Δ*ftsE* cells with 250 μM MP265 did not produce any obvious morphological changes when observed by phase contrast microscopy, however, likely due to severe growth inhibition. We therefore used treatment with 5 μM MP265 or A22 to interrogate the role of MreB in the formation and morphology of the thin connections in *ftsE* mutant cells.

**Fig 8:**
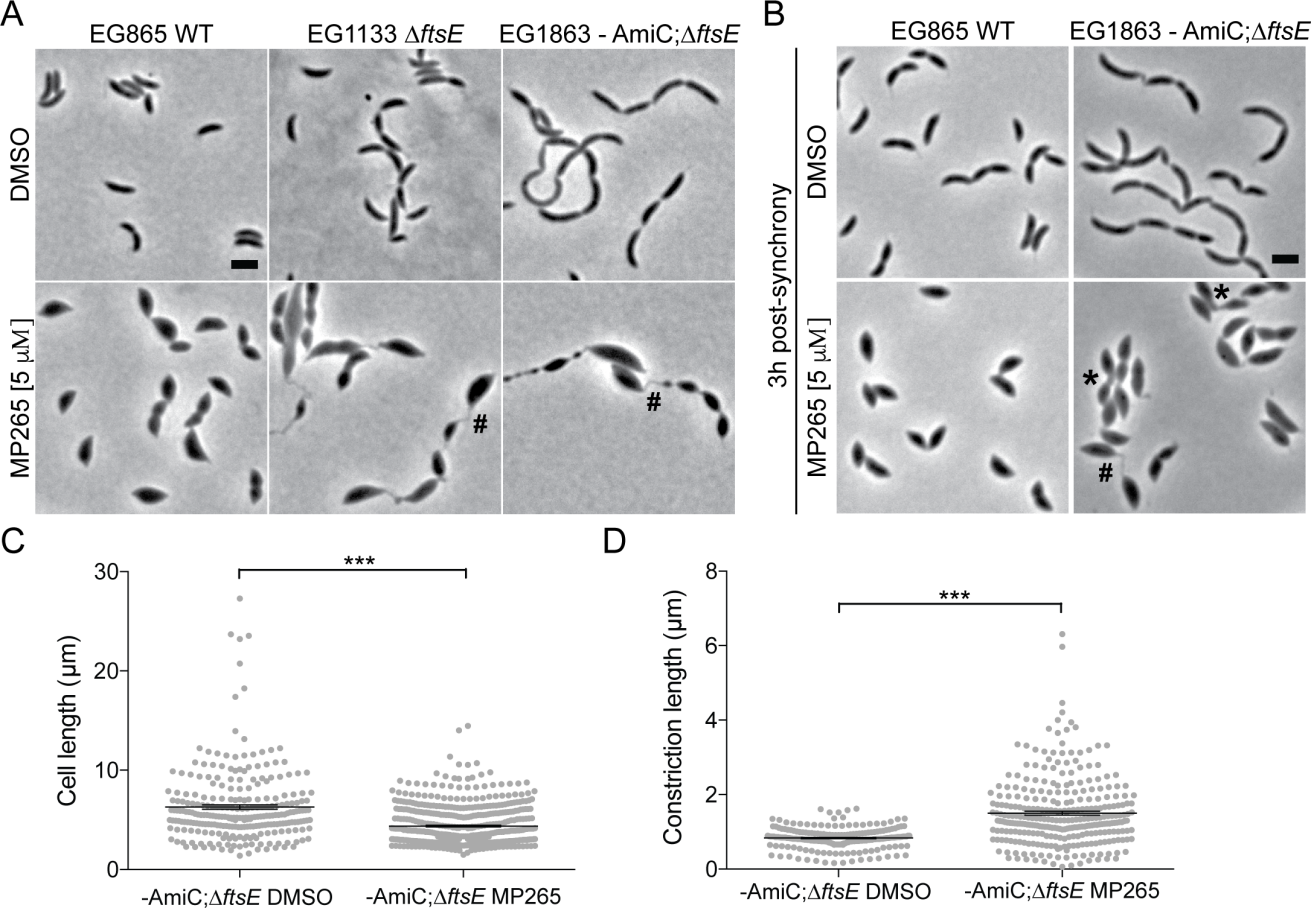
MreB inhibition leads to elongation and variable width of the thin connections in *ΔftsE* mutants. (A) Phase contrast micrographs of mixed populations of WT, *ΔftsE*, or *ΔftsE* cells depleted for AmiC (4.5 h) and treated with DMSO or 5 μM MP265 for 4.5 h. (B) Phase contrast micrographs of synchronized WT or *ΔftsE* cells depleted for AmiC and treated with DMSO or 5 μM MP265 for 3 h post-synchrony prior to imaging. AmiC was depleted for 1–1.5 h pre-synchrony and for an additional 3 h post-synchrony upon addition of DMSO or MP265. (C) Cell length distributions with mean ± SEM of strains in (B). Mean cell length (μm) ± SEM: -AmiC; *ΔftsE* DMSO = 6.29 ± 0.23 (N = 261); -AmiC; *ΔftsE* MP265 = 4.37 ± 0.09 (N = 509). (D) Constriction length distributions with mean ± SEM of strains in (B). Mean constriction length (μm) ± SEM: -AmiC; *ΔftsE* DMSO = 0.83 ± 0.02 (N = 212); -AmiC;*ΔftsE* MP265 = 1.51 ± 0.05 (N = 301). *** = p value < 0.001 by unpaired t test. * = cell-cell connections with increased width; # = skinny connections. Scale bars = 2 μm.

In both WT and *ftsE* mutant cells, 5 μM MP265 or A22 caused widening of cell bodies and tapered poles, resulting in a lemon-like cell shape (Fig 8A, S7A Fig) [38,41]. Consistent with reports implicating MreB in stalk formation, whole mount TEM of mixed or synchronized populations of MP265-treated WT or -AmiC; *ΔftsE* cells revealed a variety of stalk morphological defects including stalks that were wider, bulbous, and stunted compared to those in DMSO-treated cells (S8 Fig) [38]. Treatment of mixed populations of WT or -AmiC; *ΔftsE* cells with 5 μM MP265 affected stalk elongation without obvious changes in stalk morphology (S8A Fig). In contrast, addition of 5 μM MP265 to synchronized populations of WT or -AmiC; *ΔftsE* cells to assess effects of MreB inhibition on *de novo* stalk formation severely compromised stalk synthesis, width maintenance, and overall morphology (S8B and S8C Fig). Our observations are consistent with the previous conclusion that MreB plays a critical role in formation and elongation of stalks, but that pre-existing stalks largely maintain their morphology when MreB is inhibited [38].

Interestingly, after treating mixed or synchronized populations of Δ*ftsE* or–AmiC; Δ*ftsE* mutants with 5 μM A22 or MP265, the skinny connections became elongated and displayed more heterogeneous widths (Fig 8A and 8B, S7A Fig) [41]. We quantified the cell morphologies of synchronized -AmiC; *ΔftsE* cells treated with 5 μM MP265 for three hours, and found that MP265-treated cells were shorter and had longer skinny connections between cell bodies than those treated with DMSO (Fig 8B–8D). We conclude that, similar to WT stalks and cell bodies, the MreB-directed cell elongation machinery plays a role in regulating the width of the skinny connections; however, it is not required for the initiation or extension of the thin connections in *ftsE* mutants (S8 Fig) [38]. In fact, in contrast to stalks, the thin connections elongate more when MreB is inhibited. One possible interpretation of this result is that by inhibiting MreB, and consequently the cell elongation machinery, more substrate is available to other PG synthetic enzymes which then elaborate the skinny connections.

To further investigate the role of MreB in the elongation of the skinny connections, we labeled A22-treated WT or -AmiC; *ΔftsE* mutant cells with HADA to follow the spatial distribution of active PG metabolism. Interestingly, in WT cells treated with 5 μM A22, HADA label was enriched both at midcell and at the poles, implying that while MreB inhibition affects stalk morphology, new cell wall incorporation at the base of would-be stalks persists (S7B Fig). In -AmiC; *ΔftsE* mutant cells, we observed HADA incorporation at the base of and/or throughout the skinny connections (S7B Fig). This pattern of *de novo* PG synthesis is similar to what we observe in untreated *ftsE* mutant cells and suggests that MreB is not required for new cell wall incorporation at the extended constrictions (S4 Fig, S7B Fig). These data indicate that for both stalks and the thin connections of *ftsE* mutant cells, MreB is important for width maintenance, but is not absolutely required for localized cell wall synthesis.

## Discussion

**Fig 9:**
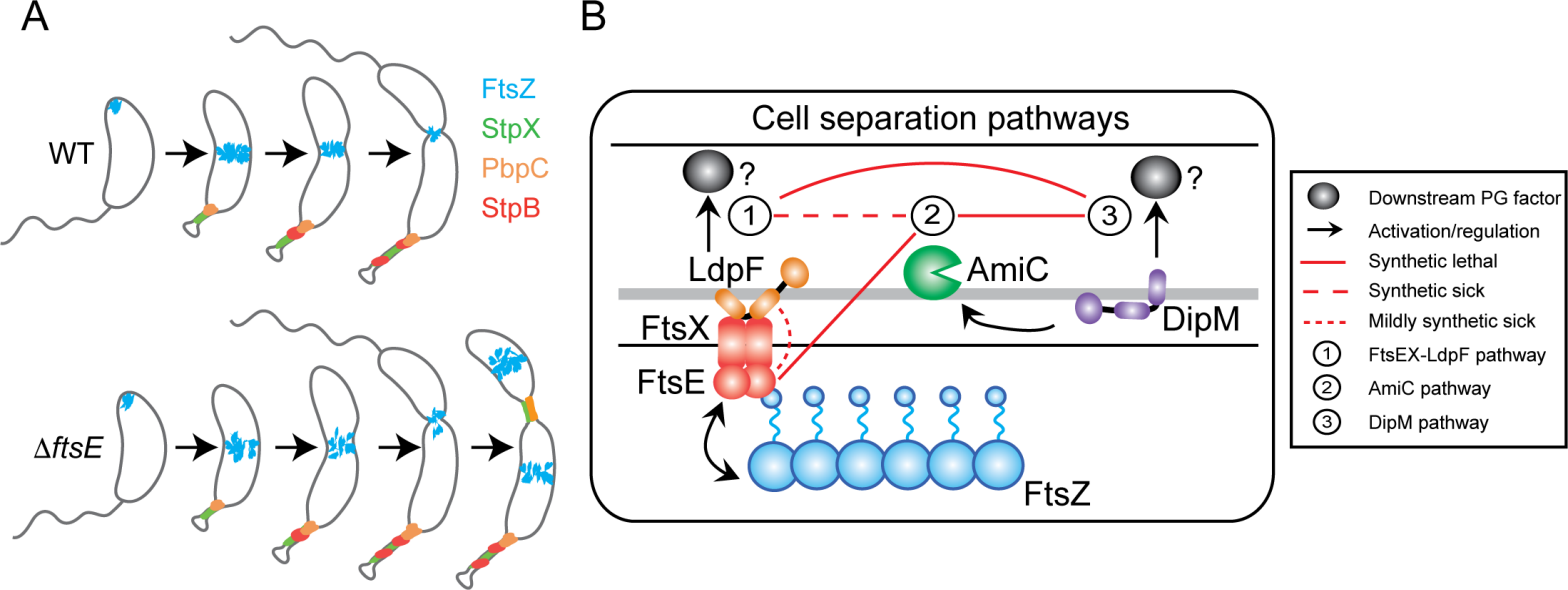
FtsEX-mediated regulation of constriction initiation and final cell separation reveals morphogenetic plasticity in *C*. *crescentus*. (A) Cell cycle localization of FtsZ and stalk proteins in WT and *ΔftsE* cells. (B) Three putative cell separation pathways in *C*. *crescentus*.

The role of FtsEX in synchronizing PG remodeling with cell division appears to be conserved amongst distantly related bacterial species such as *E*. *coli*, *S*. *pneumoniae*, and *M*. *tuberculosis*, although the downstream adaptor or enzyme targets vary [7–11]. We provide evidence that this paradigm also extends to the α-proteobacterium *C*. *crescentus*. Our data indicate that FtsE is important for initial Z-ring assembly and regulates Z-ring structure in a manner dependent on its stoichiometry with FtsX (Figs 2, 3 and 9A). Different levels of FtsE or FtsEX not only affect FtsZ localization, but also FtsZ function, namely its ability to localize incorporation of new cell wall material (Fig 3). Additionally, our data implicate FtsEX in a cell wall metabolic pathway involving LdpF and an unidentified downstream cell wall factor regulated by LdpF (Figs 4 and 9B). Thus *C*. *crescentus* FtsEX, similar to what has been proposed in *E*. *coli*, may synchronize PG remodeling with Z-ring constriction during division [9]. ECT of the skinny connections in the *ftsE* mutant revealed a cell envelope architecture remarkably distinct from *E*. *coli* hydrolase mutants, however, and an overall morphology that was strikingly stalk-like (Fig 6). Enrichment for stalk proteins StpX and PbpC at the skinny constrictions in *ΔftsE* and a strong genetic interaction between *ftsE* and the stalked cell fate determinant *spmX* further support mechanistic overlap between the elaboration of thin connections in *ftsE* mutants and stalk development (Figs 5, 7 and 9A).

During the late stages of division in *C*. *crescentus*, constriction of the inner membrane proceeds until the inner membranes of the two future daughter cell compartments are connected only by a small tubular structure [32]. Out of thousands of cells Judd and colleagues examined in their study, only five displayed inner membrane connections with diameters less than ~100 nm and the smallest inner membrane connection was 60 nm in diameter [32]. ECT of cells lacking FtsE and AmiC with fairly uniform connections showed inner membrane connection diameters ranging from ~12 to 60 nm (Fig 6). Thus, the majority of cells lacking FtsE and AmiC have inner membrane connection diameters that fall well below the lowest threshold reported for inner membrane connection diameters at any stage of WT cell division. Furthermore, WT cells spend a short amount of time in these late, transitional stages, perhaps only a few seconds, and membrane topology changes very rapidly [32]. In the case of the *ftsE* mutant cells, the thin connections are abundant in a mixed cell population and, based on their nearly abutting inner membranes, are likely blocked or delayed at a terminal stage just prior to inner membrane fission. The mechanisms underlying rapid terminal constriction and membrane fusion in bacteria are unknown [32]. Our data indicate that FtsEX is important for inner membrane fusion, either directly or indirectly, and that in the absence of FtsE, inner membrane fusion frequently fails, PG synthesis continues, and cells elaborate a stalk-like structure.

Understanding how proteins are targeted specifically to the stalk is important for understanding mechanisms of subcellular organization in bacteria as well as stalk function [37]. We have limited knowledge about the molecular pathways responsible for targeting proteins to the stalk; however, stalked pole geometry, membrane curvature, or unique peptidoglycan motifs are possible mechanisms for protein localization at the stalk. The observation that both StpX and PbpC localize to the skinny connections in *ΔftsE* cells provides an experimental handle for understanding stalk biogenesis and stalk protein localization cues. In WT *C*. *crescentus*, the bactofilins, BacA and BacB, localize at the stalked pole and recruit PbpC during the swarmer-to-stalked cell transition [36]. We have not monitored the localization of the bactofilins in *ΔftsE* cells, but we predict that they would localize at the skinny connections upstream of PbpC, similar to the protein recruitment hierarchy observed for stalks. It has been proposed that the membrane curvature at the stalk-cell body junction drives BacAB clusters to localize there [36]. These BacAB clusters could likewise recruit PbpC and StpX to the junctions between the cell bodies and skinny connections in *ΔftsE* on the basis of shared membrane curvature with the cell body-stalk junction. Additionally, the composition of the peptidoglycan is purportedly distinct between the cell body and the stalk [42], as stalks are more resistant to lysozyme treatment [43]. PbpC may regulate the rigidity of the stalk by dictating a specific cell wall remodeling regime, which may also be active at the skinny connections [36]. The periplasmic N-terminal domain of StpX is required for its stalk localization and may recognize PbpC-specific changes in PG chemistry, leading to sequestration of StpX in the stalk and the skinny constrictions of *ΔftsE* cells [35].

Within α-proteobacteria, asymmetric patterns of growth are particularly well-represented in the orders *Rhizobiales*, *Rhodobacterales*, and *Caulobacterales* [44]. Unlike *C*. *crescentus*, which divides by asymmetric binary fission, *Rhodomicrobium vannielii* and *Hyphomonas neptunium*, members of *Rhizobiales* and *Rhodobacterales* respectively, use a budding mechanism whereby new offspring emerges from the tip of a stalk-like structure [44–46]. Cell division thus occurs in an extremely asymmetric manner at the bud neck, producing a stalked mother cell and a non-stalked daughter cell [45]. After initially increasing in cell size by dispersed PG incorporation, *H*. *neptunium* displays a period of zonal growth at the new cell pole leading to the elaboration of a stalk structure [45]. This type of stalk outgrowth is similar to what occurs in other stalked α-proteobacteria like *C*. *crescentus*, which suggests conservation of core machinery. The asymmetric manner in which *H*. *neptunium* divides exemplifies how stalks may function not only as specialized organelles, but also as division planes, depending on the bacterial species [45]. The morphology of Δ*ftsE* cells, with thin, stalk-like extensions between cell bodies is reminiscent of a predivisional *H*. *neptunium* cell where the stalk is closely integrated within the cell division program.

Our data suggest that although the slender connections in *ΔftsE* share certain morphological and molecular features with stalks, they are not, in actuality, true stalks forming at failed division sites: the spatial pattern of PG incorporation is distinct from stalks, the diameters are not as homogeneous as for stalks, StpB does not localize at the skinny connections, and MreB inhibition causes the skinny connections in *ΔftsE* to elongate (Figs 6, 7 and 8, S3 Fig). Interestingly, analogous to what we observe in the *ΔftsE* thin connections, *H*. *neptunium* stalks lack cross-bands, display cell cycle dependent dispersed PG incorporation along their lengths, and become elongated at low concentrations of MP265 (5 μM) (Fig 8) [45]. Since WT *C*. *crescentus* stalks only incorporate new PG material at their base while the *ΔftsE* skinny connections and *H*. *neptunium* stalks are capable of synthesizing new PG throughout their length, cross-bands may play a role in restricting cell wall remodeling to the stalk-cell body junction [45]. Based on our MreB localization and inhibition data, it seems unlikely that the cell elongation machinery plays a primary role in initiating or extending the skinny connections, though it appears important for width control. We instead favor the hypothesis that when *ΔftsE* cells stall at a late stage in division, the division-specific PG synthetic machinery or other cell wall enzymes extend thin, stalk-like connections. We interpret this phenomenon as an example of morphogenetic plasticity, whereby small changes to established morphogenetic machineries give rise to novel or, in the case of *ΔftsE*, modified forms of preexisting structures. Overall, our findings have important implications for understanding late stage division regulation, stalk formation, and the coordination of morphogenetic events and machineries in *C*. *crescentus*. Loss of FtsE has revealed unexpected morphogenetic plasticity in *C*. *crescentus* and offers insight into the geneses of diverse morphologies in bacteria.

Note added in proof: Work from the Thanbichler lab was recently accepted that thoroughly characterizes the PG hydrolytic pathways of *C*. *crescentus*. They describe similar genetic interactions between *amiC*, *dipM*, and *ldpF* as those described here and describe additional lytic transglycosylases that function downstream of *dipM* [47].

## Materials and methods

### Growth conditions for bacterial strains

*C*. *crescentus* NA1000 strains were grown in peptone yeast extract (PYE) medium at 30°C [48]. Additives and antibiotics were used at the following concentrations in liquid (solid) media for *C*. *crescentus*: xylose 0.3 (0.3)%, glucose 0.2 (0.2)%, vanillate 0.5 (0.5) mM, gentamicin 1 (5) μg mL^-1^, kanamycin 5 (25) μg mL^-1^, spectinomycin 25 (100) μg mL^-1^, streptomycin (5 μg mL^-1^). Before changes in induction conditions, cells were washed two to three times in plain media. All MreB inhibition experiments were performed in PYE-Tris, pH 7.8 [41]. Growth rate analyses were performed in 96-well plates with shaking at 30°C using a Tecan Infinite 200 Pro plate reader. Strains and plasmids used in this study are included in S1 Table.

### Light microscopy and image analysis

Cells were imaged during the log phase of growth after immobilization on 1% agarose pads. Light microscopy was performed on a Nikon Eclipse Ti inverted microscope equipped with a Nikon Plan Fluor x 100 (numeric aperture 1.30) oil Ph3 objective and Photometrics CoolSNAP HQ cooled CCD (charge-coupled device) camera. Chroma filter cubes were used as follows: ET-EYFP for YFP and ET-ECFP for CFP, ET-dsRED for mCherry and ET-ECFP for HADA. Images were processed in Adobe Photoshop. Automated cell length analysis was performed and demographs were generated using Oufti or MicrobeJ [49,50].

### Whole cell TEM

Cells from EG864, EG1133, and EG1863 were grown in PYE and prepared for whole cell TEM exactly as described [48].

### Constriction length analysis

The length of each constriction (cell-cell connection) was determined using a homemade script in Matlab and output from Oufti [49]. Constriction segment length was defined as the length of a contiguous segment for which the width of the cell drops below 85% of the maximum cell width. The width of each cell was calculated from the Oufti output and getextradata.m from Oufti's source code was used to get the length and step length of the cell. An eight term Fourier model was fitted with cellular width as the y-axis and cellular length at which the width was calculated as the x-axis. This was done to allow for interpolation, and to adjust for small local fluctuations in width. An adjusted R-squared cutoff of 0.975 was used for fit quality before proceeding to the next step. The maximum width was then determined by finding the global maxima at least 0.2 μm away from the cell poles. The length for which the width drops below 85% of the maximum width was then calculated for each cell across each population.

### HADA labeling

Cells from strains EG379, EG864, EG930, EG1133, EG1252, EG1272, and EG1863 were grown in PYE. HADA [21] was added to ~0.41 mM and the cultures were returned to the shaker for 5 min. The cells were then washed twice with PBS and resuspended in PBS before imaging [48].

### Immunoblotting

Cells from strains EG2025 and EG2026 were immunoblotted as described in [15]. RFP antibody was used at a 1:5,000 dilution while SpmX antiserum [29] was used at a 1:10,000 dilution.

### Bacterial two-hybrid analysis

The T18 and T25 plasmids were co-transformed into BTH101 (*F-*, *cya-99*, *araD139*, *galE15*, *galK16*, *rpsL1 (Str* ^*r*^*)*, *hsdR2*, *mcrA1*, *mcrB1*; Euromedex) competent cells, plated onto LB agar with ampicillin (100 μg/μL) and kanamycin (50 μg/μL), and incubated overnight at 30°C. Several colonies were inoculated into LB with ampicillin (100 μg/μL), kanamycin (50 μg/μL), and IPTG (0.5 mM) and incubated at 30°C overnight. The next morning 2 μL of each culture was spotted onto plates containing ampicillin, kanamycin, X-gal (40 μg/mL), and IPTG (0.5 mM) and incubated for 1–2 days at 30°C. Positive interactions were indicated by blue colonies. Every interaction was tested in triplicate.

### Electron cryotomography (ECT)

For ECT imaging, strain EG1863 was grown in PYE with xylose. Once in log phase, EG1863 was washed twice with PYE and resuspended in PYE without xylose to deplete AmiC. EG1863 was then grown in PYE without xylose for ~6 h, transferred to an eppendorf tube, and shipped on ice to Grant Jensen’s lab at Caltech. The total amount of time the cells spent on ice was ~24 h. We imaged and monitored CFUs of EG1863 cells before shipment and after 24 h of incubation on ice and observed similar growth and morphology. Upon arrival, 1 mL of the EG1863 culture was centrifuged at 3000 rpm for 5 min and resuspended in fresh PYE to a final OD_600_ of ~8. This resuspension was mixed with fiducial markers (10 nm gold beads treated with bovine serum albumin to prevent aggregation) and 2 μL of the resuspension mixture was plunge-frozen on EM grids in a mixture of liquid ethane and propane [51]. Images were acquired using a 300 keV Polara transmission electron microscope (FEI) equipped with a GIF energy filter (Gatan) and a K2 Summit direct detector (Gatan). Tilt-series were collected from -50° to +50° in 1° increments at magnification of 22,500X using UCSF Tomography software [52] with a defocus of -12 μm and total dosage of 180 e^-^/Å^2^. Tomograms were calculated using IMOD software [53].

### Protein purification

Rosetta(DE3)pLysS *E*. *coli* cells containing overexpression plasmids for AmiC, LdpF, DipM, and truncated LytM domain protein variants, all with a His_6_-SUMO tag fused to the N-terminus, were purified as described previously with minor changes [15]. Rosetta cells containing the constructs were grown in 1 L of LB at 30°C to an OD_600_ of 0.4 and then induced with 1 mM IPTG for 4 h. Cells were collected by centrifugation at 6000 x g at 4°C for 10 minutes and resuspended in 40 ml Column Buffer A (CBA: 50 mM Tris-HCl pH 8.0, 300 mM NaCl, 10% glycerol, 20 mM imidazole) per 1 L of culture. Cells were snap-frozen in liquid nitrogen and stored at -80°C until use. Pellets were thawed at 37°C and lysozyme was added to 1 μg/mL and MgCl_2_ to 2.5 mM. Cell suspensions were left on ice for 45 minutes, then sonicated and centrifuged for 30 minutes at 15,000 x g at 4°C. The protein supernatant was filtered and loaded onto a HisTrap FF 1 mL column (GE Life Sciences) pre-equilibrated with CBA. The protein was eluted with 30% Column Buffer B (same as CBA except with 1M imidazole). The protein fractions were combined and His_6_-Ulp1 (SUMO protease) was added (1:500 Ulp1:protein molar ratio). The protease and protein fractions were dialyzed overnight at 4°C into CBA. Cleaved protein was run over the same HisTrap FF 1mL column equilibrated in CBA and the flow-through was collected. Flow-through fractions were dialyzed overnight at 4°C into Storage Buffer (50 mM HEPES-NaOH pH 7.2, 150 mM NaCl, 10% glycerol). Dialyzed protein was then concentrated (if needed), snap-frozen in liquid-nitrogen, and stored at -80°C.

### RBB labeled sacculi preparation

Sacculi were prepared from strain EG865 as described in [24]. *C*. *crescentus* cells were grown in 1 L of PYE at 30°C, collected at an OD_600_ of 0.6 by centrifugation at 6,000 x g for 10 minutes, and resuspended in 10 mL of PBS. The cell suspension was added drop wise to 80 mL of boiling 4% sodium dodecyl sulfate (SDS) solution. Cells were boiled and mixed for 30 minutes and then incubated overnight at room temperature. Sacculi were then pelleted by ultra-centrifugation at ~80,000 x g for 60 minutes at 25°C. Pelleted sacculi were then washed four times with ultra-pure water and resuspended in 1 mL of PBS and 20 μL of 10 mg/mL amylase and incubated at 30°C overnight. The next day, sacculi were pelleted at ~400,000 x g for 15 minutes at room temperature, washed three times with ultra-pure water, and resuspended in 1 mL of water. The sacculi suspension was labeled with 0.4 mL of 0.2 M remazol-brilliant blue (RBB), 0.3 mL 5 M NaOH, and 4.1 mL of water, and incubated at 30°C overnight. The labeled solution was neutralized with 0.4 mL of 5 M HCl and 0.75 mL of 10X PBS. Labeled sacculi were pelleted at 16,000 x g for 20 minutes at room temperature. The pellet was washed with water until the supernatant was clear. Blue-labelled sacculi were resuspended in 1 mL of 0.2% azide, incubated at 65°C for 3 hours, and then stored at 4°C.

### Dye-release assay

The dye release assay was adapted from [24]. Briefly, 10 μL of RBB-labeled sacculi was incubated at 30°C for 3 hours with AmiC, LdpF variants, DipM variants, or FtsX ECL singly or in combination. All proteins were used at 4 μM. Total reaction volumes were brought to 100 μL with PBS. Lysozyme (4 μM) was used as a positive control. After 3 hours of incubation, reactions were heat inactivated at 95°C for 10 minutes and centrifuged for 20 minutes at 16,000 x g. Supernatants were collected and the absorbance was measured at OD_595_.

## Supporting information

S1 Fig *In vivo* LdpF-mCherry has a diffuse, patchy localization in WT and *ΔftsE* cells. (A) Predicted domain organization of *E*. *coli* EnvC and *C*. *crescentus* LdpF. (B) Localization of LdpF-mCherry induced for 4 h in WT or *ΔftsE* cells. (C) α-RFP immunoblot of strains from (B). LdpF-mCherry levels are ~40% lower in *ΔftsE* compared to WT. SpmX was used as a loading control. (D) Spot dilutions of strains expressing LdpF-mCherry tagged from the native locus. Depletion strains were grown for 19 h without inducer before spotting on a PYE agar plate also without inducer. Cells in log phase were diluted to an OD_600_ of 0.05, serially diluted and spotted onto a PYE agar plate, and incubated at 30°C for 2 days. Abbreviations are as follows: SS = signal sequence; CC = coiled coil domain; LytM = LytM domain. Scale bar = 2 μm.
(TIF)

S2 Fig LdpF binds the extracellular loop (ECL) of FtsX but does not activate AmiC *in vitro*. (A) Bacterial two-hybrid analysis of T18 and T25 fusions to the ECL of FtsX, the coiled coil domain (CC) of LdpF, and the CC cytoplasmic protein ZauP, which was used as a negative control. (B,C) Dye release assay with RBB-labeled sacculi and purified variants of AmiC, LdpF, DipM, and the ECL of FtsX. Each protein was used at 4 μM and reactions were incubated at 30°C for 3 h. Reactions were performed in triplicate. *** = p < 0.0001 by one-way ANOVA.
(TIF)

S3 Fig Electron cryotomography (ECT) of cells lacking FtsE and AmiC reveal stalk-like connections of heterogeneous widths. (A-B) Tomogram slices of WT or -AmiC; Δ*ftsE* cells. (A) -AmiC; *ΔftsE* mutant skinny connection that is stalk-like, but has regions with heterogeneous widths. (B) WT *C*. *crescentus* stalks with cross-bands. # = cross-band. Scale bar (A) = 100 nm; Scale bars (B) = 200 nm.
(TIF)

S4 Fig *ftsE* mutants incorporate new cell wall material throughout skinny connections between cell bodies. HADA labeling of (A) WT, (B) *ΔftsE*, and (C) *ΔftsE* cells depleted of AmiC for 6 h. (D) FtsZ-CFP localization after 1 h of induction in *ΔftsE* cells. * = HADA incorporation throughout skinny connections in *ΔftsE*; # = absence of FtsZ at skinny connections in *ΔftsE*. Scale bars = 2 μm.
(TIF)

S5 Fig Overproducing LdpF, the LytM domain (LytM) of LdpF or AmiC is not lytic *in vivo*. Phase contrast images of cells bearing an empty vector or overproducing LdpF, the LytM domain of LdpF or AmiC for 24 h. Scale bar = 2 μm.
(TIF)

S6 Fig Treatment of WT or *ΔftsE* cells with 5 or 250 μM MP265 partially or completely arrests growth and delocalizes Venus-MreB. (A) Phase contrast and merged images of WT or *ΔftsE* cells producing Venus-MreB for 2 h. (B) Growth curves of WT, *ΔftsE*, or *ΔftsE* cells depleted for AmiC in the presence of DMSO or 5 or 250 μM MP265. Both AmiC depletion and DMSO or MP265 treatment started at the beginning of the growth curve. (C) Phase contrast and merged images of WT or *ΔftsE* cells producing Venus-MreB for 2 h. DMSO or 5 or 250 μM MP265 were added to liquid cultures for 15 min and to the agarose pads used for imaging. Scale bars = 2 μm.
(TIF)

S7 Fig New PG synthesis localizes at the skinny connections in *ΔftsE* mutants and at cell poles in WT and *ΔftsE* mutants when MreB is inhibited. (A) Phase contrast micrographs of WT, *ΔftsE*, or *ΔftsE* cells depleted for AmiC and treated with DMSO or 5 μM A22 for 4.5 h. (B) HADA labeling of WT and *ΔftsE* cells depleted of AmiC and treated with 5 μM A22 for 4 h. * = presence of HADA in *ΔftsE* skinny connections; # = polar enrichment of HADA. Scale bars = 2 μm.
(TIF)

S8 Fig Whole mount transmission electron microscopy (TEM) of MP265-treated WT or *ΔftsE* cells depleted for AmiC. (A) Micrographs of mixed populations of WT or *ΔftsE* cells depleted of AmiC and treated with 5 μM MP265 for 2.5 h. AmiC was pre-depleted for 1.5 h and for an additional 2.5 h upon addition of MP265. Micrographs of synchronized WT (B) or *ΔftsE* cells depleted of AmiC (C) treated with DMSO or 5 μM MP265 for 2 h post-synchrony. AmiC was depleted for 1.5 h pre-synchrony and for an additional 2 h post-synchrony upon addition of DMSO or MP265. * = aberrant stalk morphology. Scale bars = 500 nm.
(TIF)

S1 Text Supporting results and discussion describing biochemical investigation of cell wall hydrolase activities of LytM proteins and AmiC. (DOCX)

S1 Table Strains and plasmids used in this study with their methods of construction. (XLSX)
