# Supplementary figures and images for: FtsEX-mediated regulation of the final stages of cell division reveals morphogenetic plasticity in Caulobacter crescentus
Source: PLoS Genet. 2017 Sep 8;13(9):e1006999. doi: 10.1371/journal.pgen.1006999 (PMC5607218; doi:10.1371/journal.pgen.1006999)

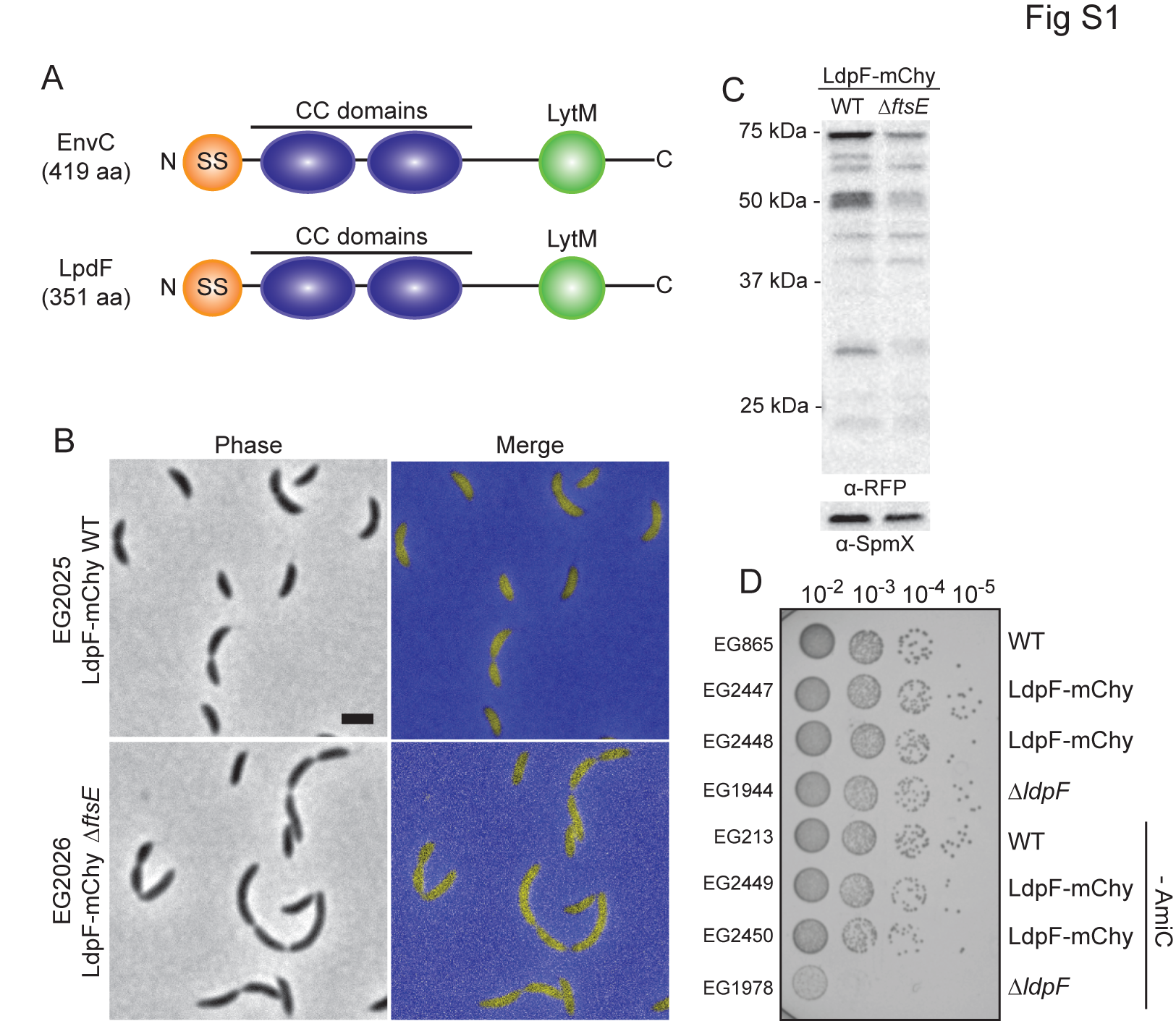

Supplement: S1 Fig — (A) Predicted domain organization of E. coli EnvC and C. crescentus LdpF. (B) Localization of LdpF-mCherry induced for 4 h in WT or ΔftsE cells. (C) α-RFP immunoblot of strains from (B). LdpF-mCherry levels are ~40% lower in ΔftsE compared to WT. SpmX was used as a loading control. (D) Spot dilutions of strains expressing LdpF-mCherry tagged from the native locus. Depletion strains were grown for 19 h without inducer before spotting on a PYE agar plate also without inducer. Cells in log phase were diluted to an OD600 of 0.05, serially diluted and spotted onto a PYE agar plate, and incubated at 30°C for 2 days. Abbreviations are as follows: SS = signal sequence; CC = coiled coil domain; LytM = LytM domain. Scale bar = 2 μm. (TIF) [file pgen.1006999.s001.tif]

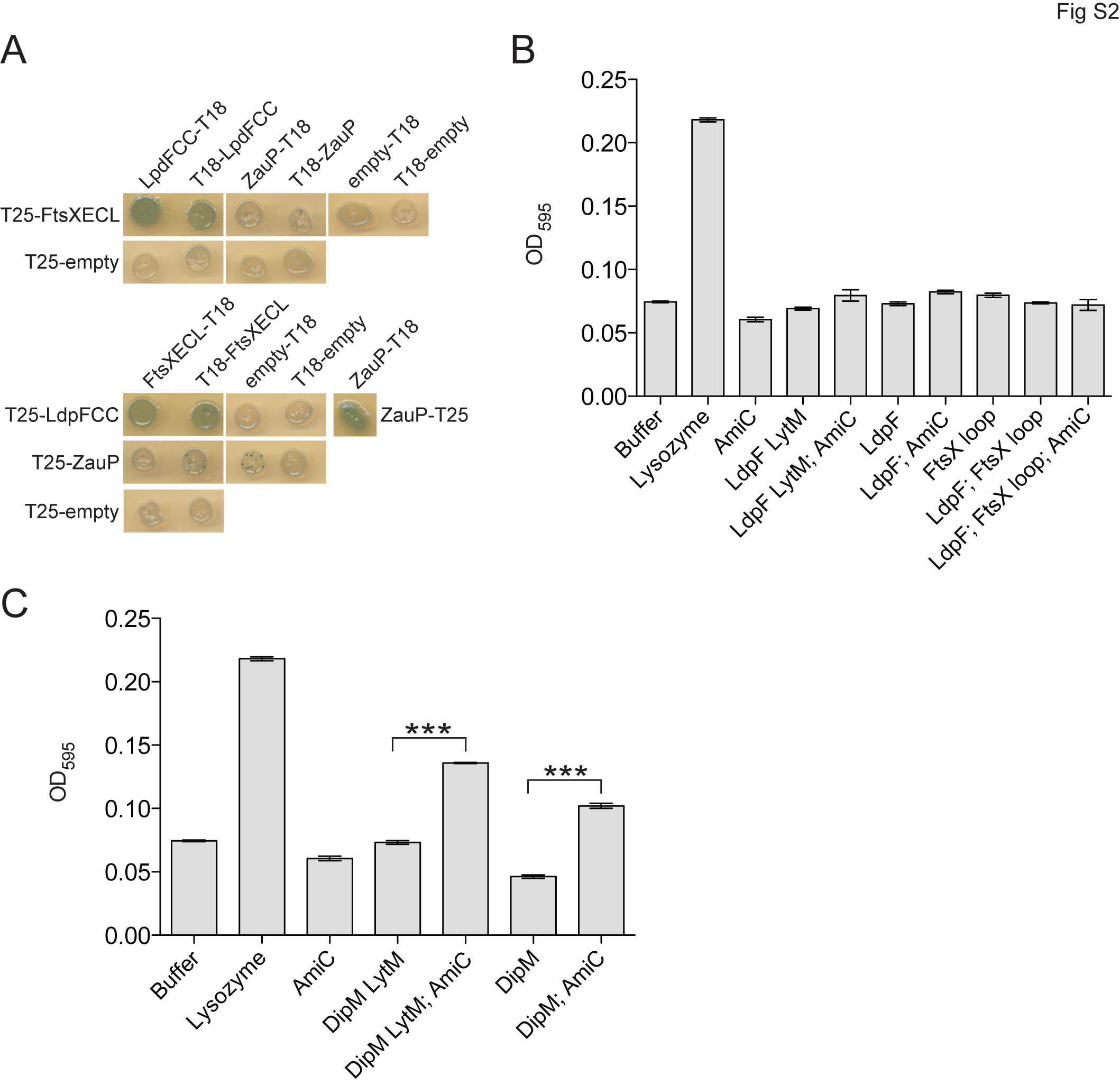

Supplement: S2 Fig — (A) Bacterial two-hybrid analysis of T18 and T25 fusions to the ECL of FtsX, the coiled coil domain (CC) of LdpF, and the CC cytoplasmic protein ZauP, which was used as a negative control. (B,C) Dye release assay with RBB-labeled sacculi and purified variants of AmiC, LdpF, DipM, and the ECL of FtsX. Each protein was used at 4 μM and reactions were incubated at 30°C for 3 h. Reactions were performed in triplicate. *** = p < 0.0001 by one-way ANOVA. (TIF) [file pgen.1006999.s002.tif]

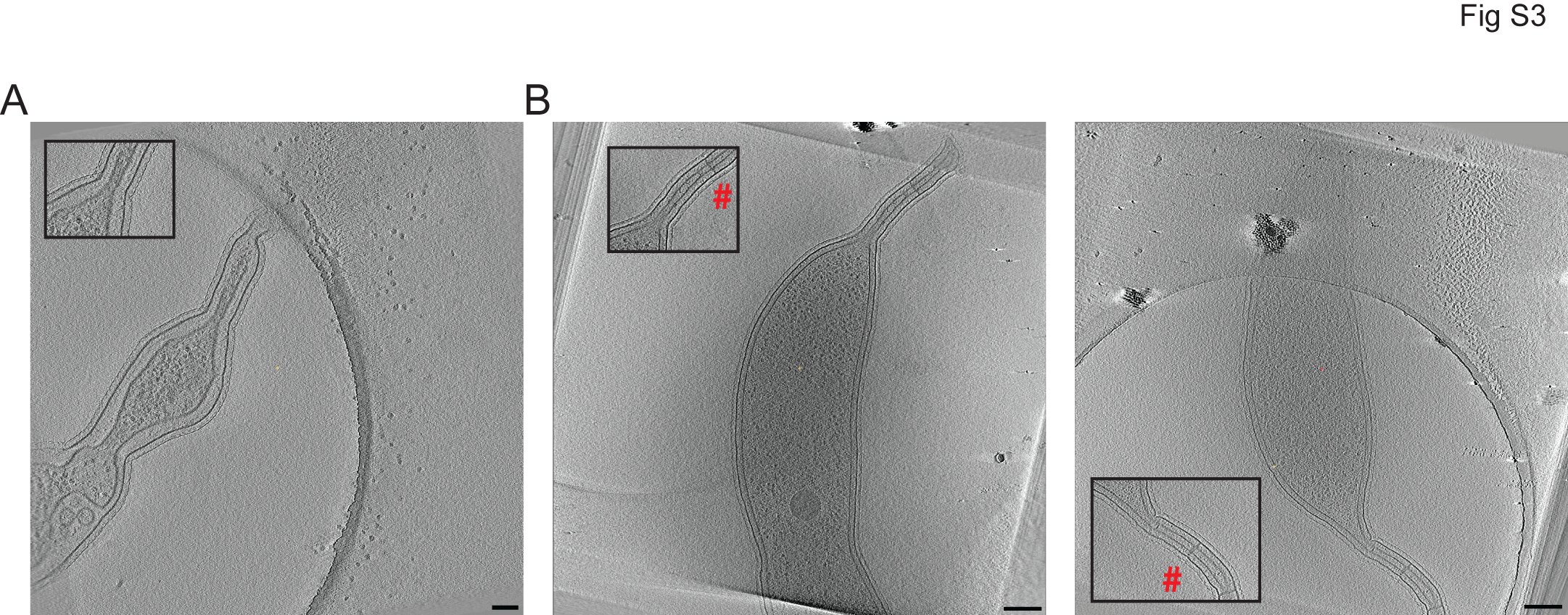

Supplement: S3 Fig — (A-B) Tomogram slices of WT or -AmiC; ΔftsE cells. (A) -AmiC; ΔftsE mutant skinny connection that is stalk-like, but has regions with heterogeneous widths. (B) WT C. crescentus stalks with cross-bands. # = cross-band. Scale bar (A) = 100 nm; Scale bars (B) = 200 nm. (TIF) [file pgen.1006999.s003.tif]

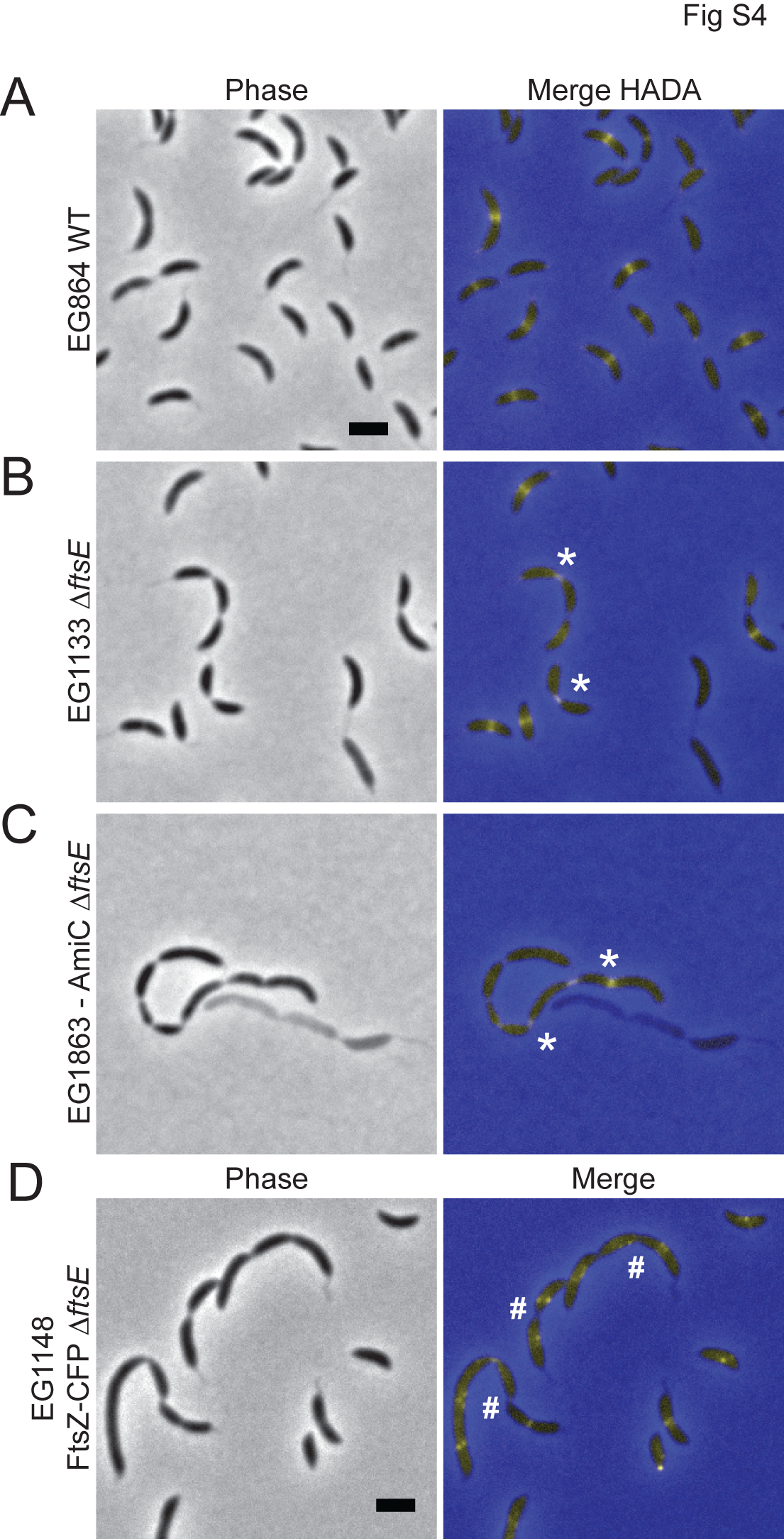

Supplement: S4 Fig — HADA labeling of (A) WT, (B) ΔftsE, and (C) ΔftsE cells depleted of AmiC for 6 h. (D) FtsZ-CFP localization after 1 h of induction in ΔftsE cells. * = HADA incorporation throughout skinny connections in ΔftsE; # = absence of FtsZ at skinny connections in ΔftsE. Scale bars = 2 μm. (TIF) [file pgen.1006999.s004.tif]

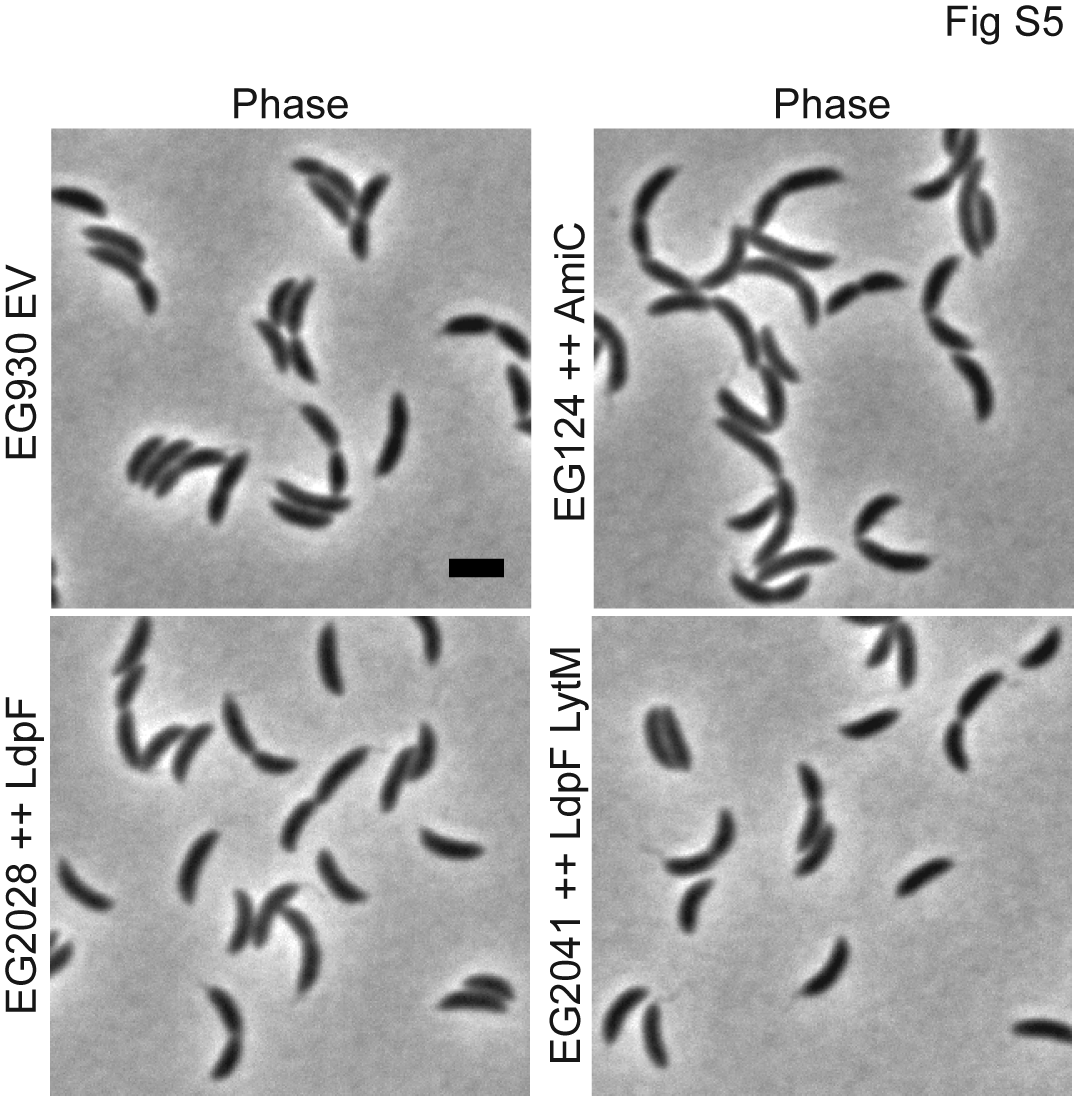

Supplement: S5 Fig — Phase contrast images of cells bearing an empty vector or overproducing LdpF, the LytM domain of LdpF or AmiC for 24 h. Scale bar = 2 μm. (TIF) [file pgen.1006999.s005.tif]

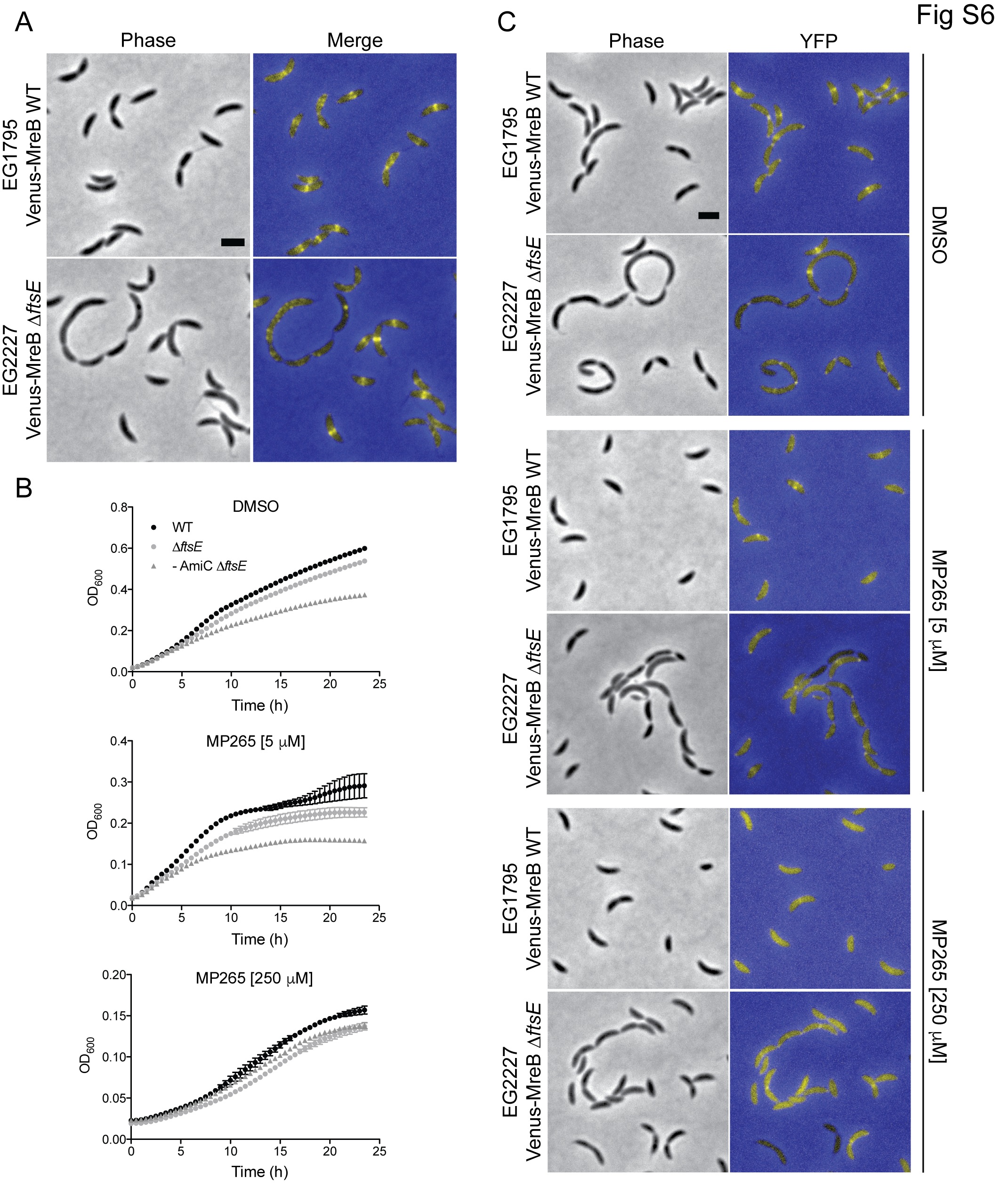

Supplement: S6 Fig — (A) Phase contrast and merged images of WT or ΔftsE cells producing Venus-MreB for 2 h. (B) Growth curves of WT, ΔftsE, or ΔftsE cells depleted for AmiC in the presence of DMSO or 5 or 250 μM MP265. Both AmiC depletion and DMSO or MP265 treatment started at the beginning of the growth curve. (C) Phase contrast and merged images of WT or ΔftsE cells producing Venus-MreB for 2 h. DMSO or 5 or 250 μM MP265 were added to liquid cultures for 15 min and to the agarose pads used for imaging. Scale bars = 2 μm. (TIF) [file pgen.1006999.s006.tif]

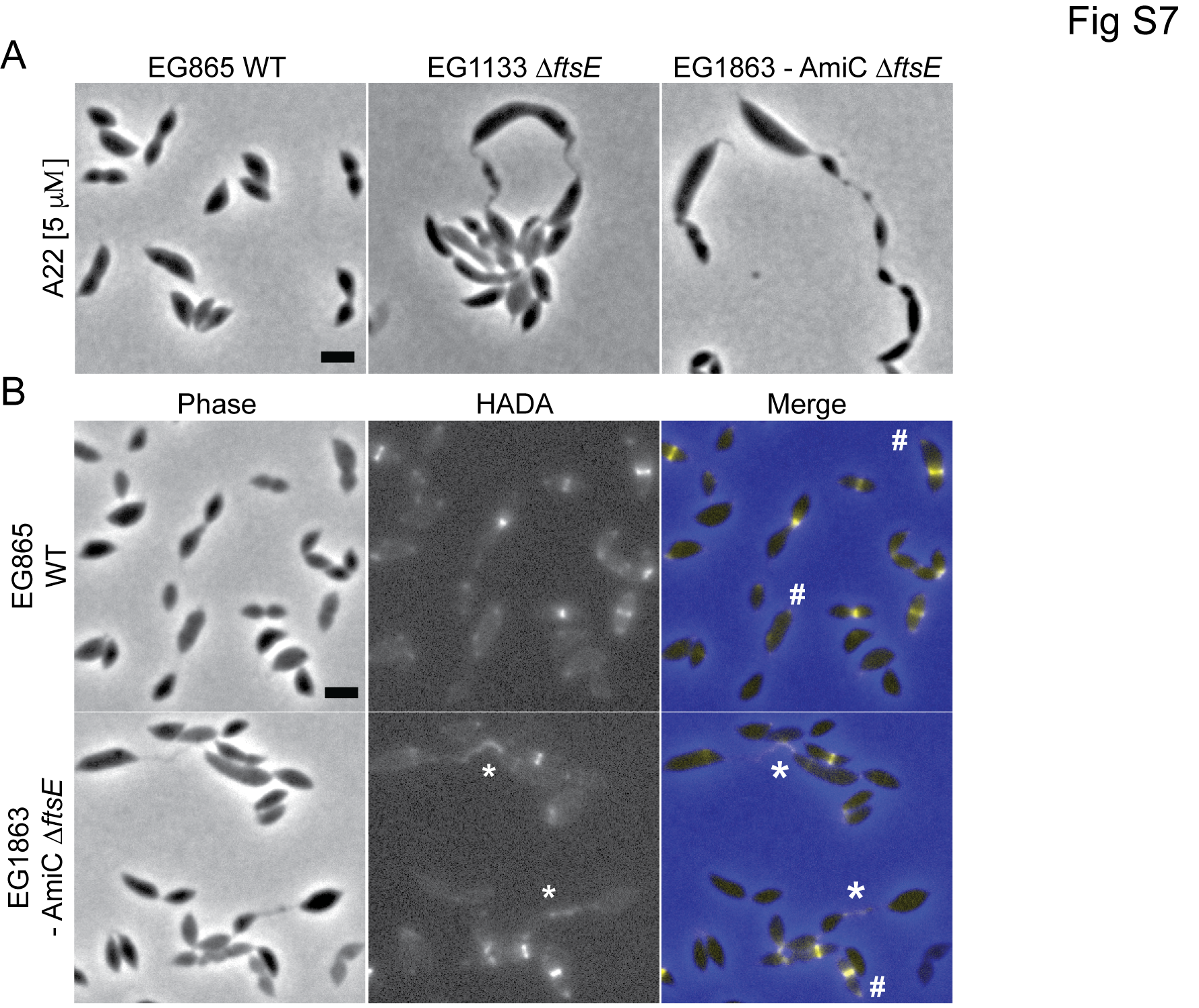

Supplement: S7 Fig — (A) Phase contrast micrographs of WT, ΔftsE, or ΔftsE cells depleted for AmiC and treated with DMSO or 5 μM A22 for 4.5 h. (B) HADA labeling of WT and ΔftsE cells depleted of AmiC and treated with 5 μM A22 for 4 h. * = presence of HADA in ΔftsE skinny connections; # = polar enrichment of HADA. Scale bars = 2 μm. (TIF) [file pgen.1006999.s007.tif]

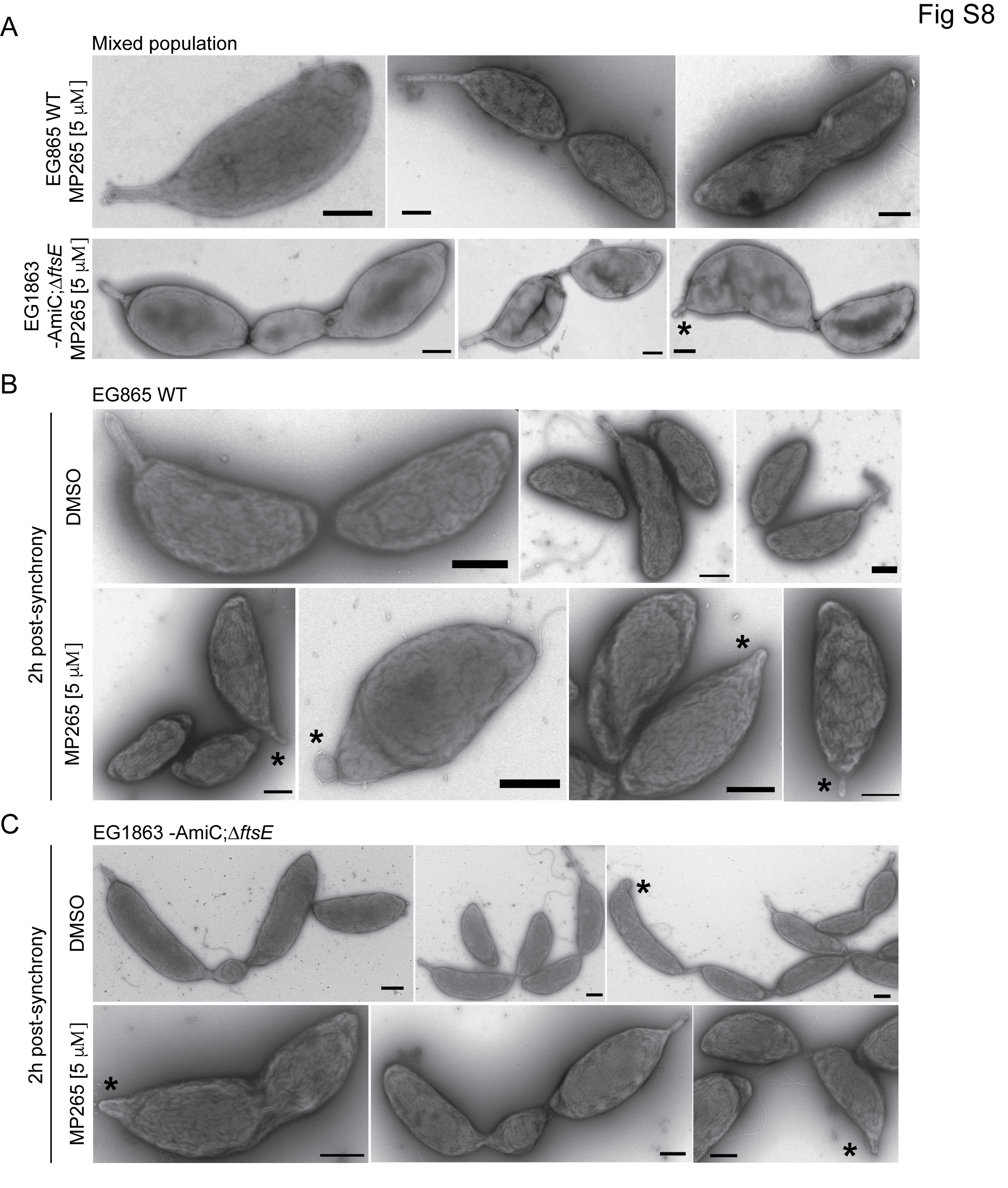

Supplement: S8 Fig — (A) Micrographs of mixed populations of WT or ΔftsE cells depleted of AmiC and treated with 5 μM MP265 for 2.5 h. AmiC was pre-depleted for 1.5 h and for an additional 2.5 h upon addition of MP265. Micrographs of synchronized WT (B) or ΔftsE cells depleted of AmiC (C) treated with DMSO or 5 μM MP265 for 2 h post-synchrony. AmiC was depleted for 1.5 h pre-synchrony and for an additional 2 h post-synchrony upon addition of DMSO or MP265. * = aberrant stalk morphology. Scale bars = 500 nm. (TIF) [file pgen.1006999.s008.tif]
