# Supplementary material for: FtsEX-mediated regulation of the final stages of cell division reveals morphogenetic plasticity in Caulobacter crescentus
Source: PLoS Genet. 2017 Sep 8;13(9):e1006999. doi: 10.1371/journal.pgen.1006999 (PMC5607218; doi:10.1371/journal.pgen.1006999)
Supplement: S1 Text — (DOCX) [file pgen.1006999.s009.docx]

**FtsEX-mediated regulation of the final stages of cell division reveals morphogenetic plasticity in *Caulobacter crescentus***

**Meier et al.**

**S1 Text**

**The CC domain of LdpF interacts with the ECL of FtsX, but LdpF does not activate AmiC for PG hydrolysis *in vitro***

Since the EnvC-FtsX interaction is mediated by contacts between the first periplasmic loop of FtsX and the coiled-coil (CC) domain of EnvC [1], we tested for an interaction between the CC domain of LdpF and the ECL of FtsX. Bacterial two hybrid analysis showed a positive result for the CC domain of LdpF and the ECL of FtsX, but not for the highly CC cytoplasmic division protein ZauP and the ECL of FtsX, suggesting that the ECL of FtsX interacts with the CC domain of LdpF specifically and not CC domains generally (S2 Fig). Based on the positive interaction observed for the CC domain of LdpF and the ECL of FtsX, we next attempted to use a previously established dye-release assay to reconstitute PG hydrolase activity [2,3]. The LytM domain of EnvC is sufficient to activate AmiA/B while full length NlpD activates AmiC to hydrolyze PG *in vitro* [3]. We therefore purified *C. crescentus* full length AmiC, full length LdpF, the LytM domain of LdpF, full length DipM, the LytM domain of DipM, and FtsX ECL and assayed proteins individually or in combination for their ability to degrade PG. We labeled the sugar moieties of purified *C. crescentus* sacculi with remazol brilliant blue (RBB) and incubated the RBB-labeled PG and purified protein(s) at 30^o^C. After 3 hours, we terminated the reactions and measured the dye remaining in the supernatant after pelleting uncleaved PG [3]. Our positive control, lysozyme, displayed robust hydrolysis activity, however none of the proteins individually hydrolyzed PG to an appreciable extent compared to buffer alone (S2 Fig). Although LdpF was unable to activate AmiC hydrolytic activity, reactions containing AmiC and either full length DipM or its LytM domain displayed moderate hydrolase activity *in vitro*, which is consistent with the shared homology between DipM and NlpD and *C. crescentus* AmiC and *E. coli* AmiC. In *E. coli*, low level production of the LytM domain of EnvC is sufficient to induce toxicity and cell lysis and in *C. crescentus*, overproduction of DipM also leads to cell lysis [3-5]. Overproducing AmiC, full length LdpF, or the LytM domain of LdpF for 24 hours, however, did not induce lysis, which suggests that *C. crescentus* AmiC may only have weak hydrolytic activity *in vivo* and/or the downstream target of LdpF may not be AmiC, or even a hydrolase for that matter (S5 Fig). Given the genetic interactions between FtsE and SpmX and the fact that SpmX contains a muramidase domain, we also tested for LdpF-mediated activation of hydrolytic activity by SpmX using the dye release assay. However, we did not observe any activity for SpmX with or without LdpF.

At high protein concentrations (4 uM) and long incubation times (overnight), LdpF alone weakly hydrolyzed PG, however we are unconvinced that this activity is physiologically relevant. There are a number of reasons that may explain why we did not observe PG hydrolysis in our *in vitro* setup. Since we only tested the effect of the ECL of FtsX in our dye release assay, we cannot discount the possibility that the reconstituted FtsEX complex is required for LdpF-mediated AmiC activation *in vitro*. *S. pneumoniae* PcsB +/- the ECL of FtsX lacks *in vitro* enzymatic activity which raises the possibility that activation of PG hydrolysis by PcsB may require additional factors besides the ECL of FtsX and/or conformational changes arising from ATP hydrolysis by FtsE [7]. Analogously, a critical, unknown factor may be absent from our *in vitro* assay, which would explain our lack of observed activity. It may also be that LdpF does not activate an amidase, or hydrolase, at all, but instead regulates some other aspect of PG metabolism.

**Supporting Information References**

1. Yang DC, Peters NT, Parzych KR, Uehara T, Markovski M, Bernhardt TG. An ATP-binding cassette transporter-like complex governs cell-wall hydrolysis at the bacterial cytokinetic ring. Proc Natl Acad USA. 2011;108(45): E1052-60. doi: 10.1073/pnas.1107780108.
2. Zhou R, Chen S, Recsei P. A dye release assay for determination of lysostaphin activity. Anal Biochem. 1988;171(1): 141-4.
3. Uehara T, Parzych KR, Dinh T, Bernhardt TG. Daughter cell separation is controlled by cytokinetic ring-activated cell wall hydrolysis. EMBO J. 2010;29(8): 1412-22. doi: 10.1038/emboj.2010.36.
4. Goley ED, Comolli LR, Fero KE, Downing KH, Shapiro L. DipM links peptidoglycan remodeling to outer membrane organization in *Caulobacter*. Mol Microbiol. 2010;77(1): 56-73. doi: 10.1111/j.1365-2958.2010.07222.
5. Moll A, Schlimpert S, Briegel A, Jensen GJ, Thanbichler M. DipM, a new factor required for peptidoglycan remodeling during cell division in *Caulobacter crescentus*. Mol Microbiol. 2010;77(1): 90-107. doi: 10.1111/j.1365-2958.2010.07224.
6. Sham LT, Barendt SM, Kopecky KE, Winkler ME. Essential PcsB putative peptidoglycan hydrolase interacts with the essential FtsXSpn cell division protein in *Streptococcus pneumonaie* D39. Proc Natl Acad Sci USA. 2011;108(45): E1061-9. doi: 10.1073/pnas.1108323108.
7. Evinger M and Agabian N. Envelope-associated nucleoid from *Caulobacter crescentus* stalked and swarmer cells. J Bacteriol. 1977;132(1): 294-301.
8. Radhakrishnan SK, Thanbichler M, Viollier PH. The dynamic interplay between a cell fate determinant and a lysozyme homolog drives the asymmetric division cycle of *Caulobacter crescentus*. Genes Dev. 2008;22(2): 212-25. doi: 10.1101/gad.1601808.
9. Meier EL, Razavi S, Inoue T, Goley ED. A novel membrane anchor for FtsZ is linked to cell wall hydrolysis in *Caulobacter crescentus*. Mol Microbiol. 2016;101(2): 265-80. doi: 10.1111/mmi.13388.
10. Woldemeskel SA, McQuillen R, Hessel AM, Xiao J, Goley ED. A conserved coiled-coil protein pair focuses the cytokinetic Z-ring in *Caulobacter crescentus*. Mol Microbiol. 2017. doi: 10.1111/mmi.13731. [Epub ahead of print]
11. Thanbichler M and Shapiro L. MipZ, a spatial regulator coordinating chromosome segregation with cell division in *Caulobacter*. Cell. 2006;126(1): 147-62.
12. Zielinska A, Billini M, Moll A, Kremer K, Briegel A, Martinez AI, et al. LytM factors affect the recruitment of autolysins to the cell division site in *Caulobacter crescentus*. Mol Microbiol. In review.
13. Wang Y, Jodes BD, Brun YV. A set of ftsZ mutants blocked at different stages of cell division in *Caulobacter*. Mol Microbiol. 2001;40(2): 347-60.
14. Thanbichler M, Iniesta AA, Shapiro L. A comprehensive set of plasmids for vanillate- and xylose-inducible gene expression in *Caulobacter crescentus*. Nucleic Acids Res. 2007;35(20): e137.
15. Karimova G, Pidoux J, Ullmann A, Ladant D. A bacterial two-hybrid system based on a reconstituted signal transduction pathway. Proc Natl Acad Sci USA. 1998;95(10): 5752-6.
16. Bendezu FO, Hale CA, Bernhardt TG, de Boer PA. RodZ(YfgA) is required for proper assembly of the MreB actin cytoskeleton and cell shape in E. coli. EMBO J. 2009;28(3): 193-204. doi:10.1038/emboj.2008.264.
17. Sundararajan K, Miguel A, Desmarais SM, Meier EL, Casey Huang K, Goley ED. The bacterial tubulin FtsZ requires its intrinsically disordered linker to direct robust cell wall construction. Nat Commun. 2015;6: 7281. doi: 10.1038/ncomms8281.
18. Goley ED, Yeh YC, Hong SH, Fero MJ, Abeliuk E, McAdams HH, et al. Assembly of the *Caulobacter* cell division machine. Mol Microbiol. 2011;80(6): 1680-98. doi: 10.1111/j.1365-2958.2011.07677.
19. Hughes HV, Lisher JP, Hardy GG, Kysela DT, Arnold RJ, Giedroc DP, et al. Co-ordinate synthesis and protein localization in a bacterial organelle by the action of a penicillin binding-protein. Mol Microbiol. 2013;90(6): 1162-77. doi: 10.1111/mmi.12422.
20. Strobel W, Moll A, Kiekebusch D, Klein KE, Thanbichler M. Function and localization dynamics of bifunctional penicillin-binding proteins in *Caulobacter crescentus*. J Bacteriol. 2014;196(8):1627-39. doi: 10.1128/JB.01194-13.
21. Kuhn J, Briegel A, Morschel E, Kahnt J, Leser K, Wick S, et al. Bactofilins, a ubiquitous class of cytoskeletal proteins mediating polar localization of a cell wall synthase in *Caulobacter crescentus*. EMBO J. 2010;29(2): 327-39. doi: 10.1038/emboj.2009.358.
